# Std fimbriae-fucose interaction increases *Salmonella*-induced intestinal inflammation and prolongs colonization

**DOI:** 10.1371/journal.ppat.1007915

**Published:** 2019-07-22

**Authors:** Abdulhadi Suwandi, Alibek Galeev, René Riedel, Samriti Sharma, Katrin Seeger, Torsten Sterzenbach, Lucía García Pastor, Erin C. Boyle, Ohad Gal-Mor, Michael Hensel, Josep Casadesús, John F. Baines, Guntram A. Grassl

**Affiliations:** 1 Institute of Medical Microbiology and Hospital Epidemiology, Hannover Medical School and German Center for Infection Research (DZIF), Hannover, Germany; 2 Max Planck Institute for Evolutionary Biology, Evolutionary Genomics, Plön, Germany and Christian-Albrechts-University of Kiel, Kiel, Germany; 3 Division of Microbiology and CellNanOs–Center for Cellular Nanoanalytics, University of Osnabrück, Osnabrück, Germany; 4 Departamento de Genética, Facultad de Biología, Universidad de Sevilla, Sevilla, Spain; 5 Institute for Laboratory Animal Science, Hannover Medical School, Hannover, Germany; 6 Department of Cardiothoracic, Transplantation, and Vascular Surgery, Hannover Medical School, Hannover, Germany; 7 The Infectious Diseases Research Laboratory, Sheba Medical Center, Tel-Hashomer, Israel; 8 Department of Clinical Microbiology and Immunology, Sackler Faculty of Medicine, Tel Aviv University, Tel Aviv, Israel; University of California Davis School of Medicine, UNITED STATES

## Abstract

Expression of ABO and Lewis histo-blood group antigens by the gastrointestinal epithelium is governed by an α-1,2-fucosyltransferase enzyme encoded by the *Fut2* gene. Alterations in mucin glycosylation have been associated with susceptibility to various bacterial and viral infections. *Salmonella enterica* serovar Typhimurium is a food-borne pathogen and a major cause of gastroenteritis. In order to determine the role of *Fut2*-dependent glycans in *Salmonella*-triggered intestinal inflammation, *Fut2*^*+/+*^ and *Fut2*^*-/-*^ mice were orally infected with *S*. Typhimurium and bacterial colonization and intestinal inflammation were analyzed. Bacterial load in the intestine of *Fut2*^-/-^ mice was significantly lower compared to *Fut2*^+/+^ mice. Analysis of histopathological changes revealed significantly lower levels of intestinal inflammation in *Fut2*^-/-^ mice compared to *Fut2*^+/+^ mice and measurement of lipocalin-2 level in feces corroborated histopathological findings. *Salmonella* express fimbriae that assist in adherence of bacteria to host cells thereby facilitating their invasion. The *std* fimbrial operon of *S*. Typhimurium encodes the π-class Std fimbriae which bind terminal α(1,2)-fucose residues. An isogenic mutant of *S*. Typhimurium lacking Std fimbriae colonized *Fut2*^*+/+*^ and *Fut2*^-/-^ mice to similar levels and resulted in similar intestinal inflammation. *In vitro* adhesion assays revealed that bacteria possessing Std fimbriae adhered significantly more to fucosylated cell lines or primary epithelial cells in comparison to cells lacking α(1,2)-fucose. Overall, these results indicate that *Salmonella*-triggered intestinal inflammation and colonization are dependent on Std-fucose interaction.

## Author summary

The intestinal epithelium is a crucial biological interface, interacting with both commensal and pathogenic microorganisms. It’s lined with heavily glycosylated proteins and glycolipids which can act as both attachment sites and energy sources for intestinal bacteria. Fut2, the enzyme governing epithelial α1,2-fucosylation, has been implicated in the interaction between microbes and intestinal epithelial cells. *Salmonella* is one of the most important bacterial gastrointestinal pathogens affecting millions of people worldwide. *Salmonella* possesses fimbrial and non-fimbrial adhesins which can be used to adhere to host cells. Here we show that *Salmonella* expresses Std fimbriae in the gastrointestinal tract *in vivo* and exploit Std fimbriae to bind fucosylated structures in the mucus and on the intestinal epithelium. Furthermore, we demonstrate that the Std fimbriae-fucose interaction is necessary for bacterial colonization of the intestine and for triggering intestinal inflammation. These data lend new insights into bacterial adhesion-epithelial interactions which are essential for bacterial pathogenesis and key factors in determining tissue tropism and host susceptibility to infectious disease.

## Introduction

Glycosylation is an important type of post-translational modification of proteins and lipids and is involved in the regulation of a wide range of processes at the cellular and molecular level. The gastrointestinal tract is home to a vast array of glycan structures and glycoconjugates [1], where the mucosal surface is the site of complex interactions between the intestinal microbiota, intestinal barrier, and immune system. The mucosal surface is characterized by a heavily glycosylated mucus layer produced by goblet cells as well as membrane-bound glycosylated proteins and lipids that form them [2]. These glycoconjugates can be utilized by intestinal commensal bacteria and pathogens as molecular attachment sites or as nutrients [3]. Importantly, host-derived glycans can help foster beneficial relationships with symbiotic microbes, such as *Bacteroides thetaiotaomicron*, by providing an energy source in the absence of dietary polysaccharides [4].

The *FUT2* gene encodes the α-1,2-fucosyltransferase, a glycosyltransferase well known for its role in the expression of ABH and Lewis histo-blood group antigens on the gastrointestinal epithelium and in bodily secretions. Individuals expressing a functional allele are commonly described as ‘secretors’ whereas those homozygous for loss-of-function mutations display a ‘non-secretor’ phenotype. Variation in host glycosylation may directly influence susceptibility to enteric pathogens such as enterotoxigenic *Escherichia coli* [5], *Helicobacter pylori* [6], and norovirus [7]. Recent studies have shown the importance of host glycans in supporting a beneficial relationship with the endogenous microbiota by nourishing the microbiota during the stress of systemic infection [8] or by controlling opportunistic pathogens within the microbiota in the context of infection (e.g. *Enterococcus faecalis*) [9]. The non-secretor phenotype is also associated with an increased risk to develop chronic inflammatory bowel diseases [10]. This is possibly due to the altered composition of the intestinal microbiota, which may in turn influence the capacity of pathogenic bacteria to bind to host mucosal surface structures [11].

*Salmonella enterica* serovar Typhimurium (*S*. Typhimurium) is one of the most successful mucosal pathogens, colonizing the human gastrointestinal tract and causing severe inflammatory diarrhea [12]. *S*. Typhimurium carries several virulence genes including fimbrial adhesins, which are hair-like appendages on the outer membrane and are involved in adherence to host epithelial cells. Adhesion to host tissues is critical for invasion and pathogenicity of *S*. Typhimurium [13]. Type 1 fimbriae are one of the best characterized fimbrial adhesins and are encoded by the *fim* operon. FimH, a lectin-like protein, directly binds to high mannose oligosaccharides conjugated to surface glycoproteins of eukaryotic cells [14,15]. Another fimbrial operon, *std*, encodes the π-class Std fimbriae, which have been described to bind terminal α-1,2 fucose residues [16].

The expression of bacterial adhesins possibly involved in binding fucosylated host proteins suggests that these fimbriae may facilitate *Salmonella* to establish or maintain infection in the highly fucosylated large intestine. Here, we investigated the role of host fucosylation in disease development during *Salmonella* infection using mice with and without expression of the *Fut2* gene (*Fut2*^+/+^ and *Fut2*^*-/-*^*)*. Taken together, our results demonstrate that Std-fucose interaction contributes to *S*. Typhimurium persistence and inflammation.

## Results

### Fut2 expression affects susceptibility to *Salmonella-*induced colitis

To test the hypothesis that expression of *Fut2* influences host susceptibility to enteric pathogens, a model of *S*. Typhimurium-induced colitis was utilized. *Fut2*^+/+^ and *Fut2*^-/-^ littermates were treated with streptomycin, and 24 hours later, infected with wild-type *S*. Typhimurium. One day post infection (p.i.), the cecal tissue of *Fut2*^-/-^ mice contained more *S*. Typhimurium than *Fut2*^+/+^ mice (S1A Fig) in agreement with the observations of Goto and colleagues [17]. However, in contrast to their results, we found the total cecum weight and histopathology scores (S1B–S1D Fig) were comparable between *Fut2*^+/+^ and *Fut2*^-/-^ mice.

**Fig 1:**
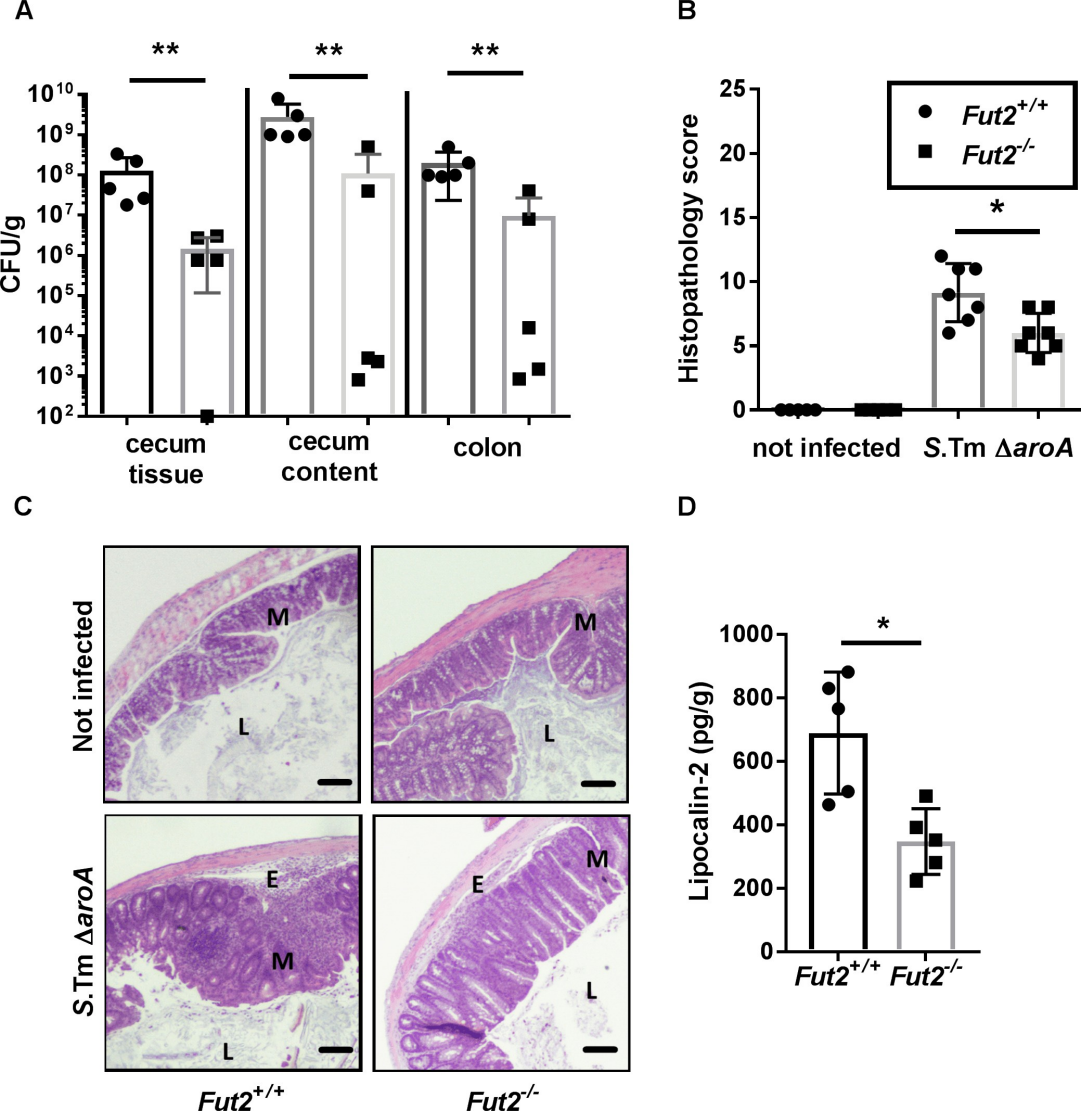
*Fut2* expression affects susceptibility to *Salmonella* induced colitis. Streptomycin-treated mice were infected with *S*. Typhimurium Δ*aroA* for 7 days. (A) *S*. Typhimurium loads were determined in cecum tissue, cecum content, and colon tissue by plating homogenates on LB agar supplemented with streptomycin (n = 5 mice per group). (B) Histology scoring revealed higher inflammation in *Fut2*^*+/+*^-infected colons in comparison to *Fut2*^*-/-*^-infected colons at day 7 post infection (p.i.). Uninfected colons of *Fut2*^*+/+*^ and *Fut2*^*-/-*^mice had low histology scores. (C) H&E staining of colon tissue sections at 7 days p.i. Scale bars, 50 μm. *Fut2*^*+/+*^ and *Fut2*^*-/-*^ uninfected mice had a normal tissue and no signs of pathology. Higher numbers of cells in the lumen (L), an increased number of inflammatory cells in mucosa (M), elevated epithelial cell desquamation, and the formation of submucosal edema (E) upon *S*. Typhimurium infection were observed in *Fut2*^*+/+*^ mice comparing to *Fut2*^*-/-*^ mice. (D) Lipocalin-2 levels measured by ELISA in supernatants of colon tissues homogenates (n = 5 per group) were higher in *Fut2*^*+/+*^ mice compared to *Fut2*^*-/-*^ mice. Graphs are representative of three independent experiments. *p<0.05; **p<0.01, Mann-Whitney test.

Under most conditions wild-type *Salmonella* kill C57BL/6 mice within approximately one week. Therefore, in order to follow the infection to later time points mice were infected with the *S*. Typhimurium *ΔaroA* mutant strain which is attenuated for systemic disease but causes extensive intestinal inflammation [18]. There was no significant difference in bacterial colonization or resulting inflammation of *Fut2*^+/+^ and *Fut2*^-/-^ mice on day 1 p.i. (S1 Fig) or on day 3 p.i. (S2A–S2C Fig). However, on day 7 and day 14 p.i., a significantly reduced *Salmonella* burden in the intestine of *Fut2*^-/-^ compared to *Fut2*^+/+^ mice was detected (Fig 1A, S2A–S2C Fig). Furthermore, 7 days p.i., the histopathological changes in the colon were significantly less severe in *Fut2*^-/-^ mice compared to *Fut2*^+/+^ animals (Fig 1B and 1C). Notably, the colons of infected *Fut2*^+/+^ mice were characterized by a higher number of detached epithelial cells within the colon lumen, increased inflammatory cell infiltration within the mucosa, and stronger submucosal edema. Additionally, the levels of the inflammation-associated marker lipocalin-2 were quantified in the large intestine after *S*. Typhimurium infection. The concentration of lipocalin-2 in the colon and cecum 7 days p.i. were significantly higher in *Fut2*^+/+^ mice in comparison to *Fut2*^-/-^ (Fig 1D, S2D Fig).

**Fig 2:**
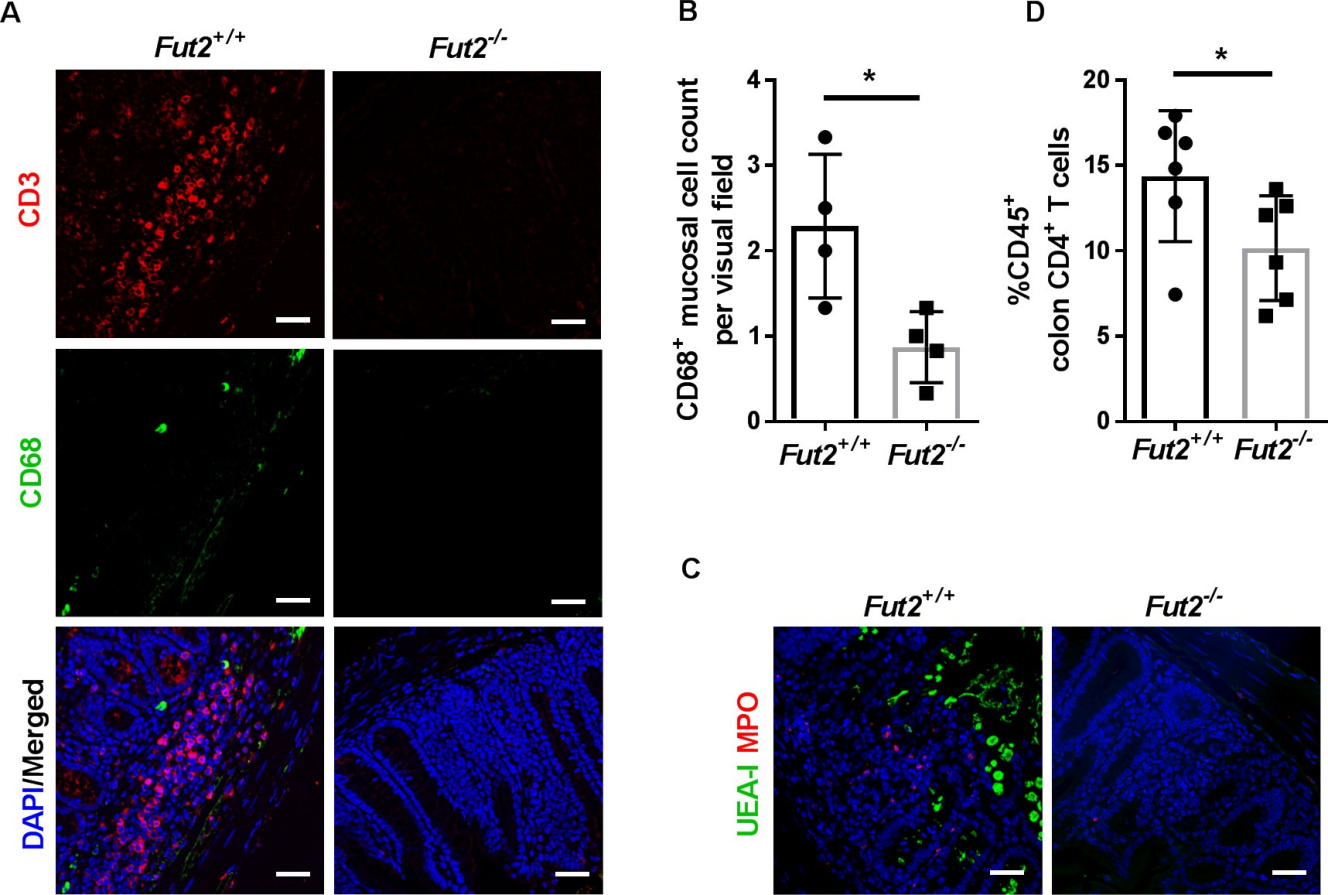
Increased infiltration of immune cells in *Fut2-*expressing mice after *Salmonella* infection. (A) Immunofluorescence staining showed that *Fut2*^*+/+*^ mice have higher numbers of CD3- (red) and CD68- (green) positive cells in the colon mucosa at day 7 p.i. Nuclei were stained with DAPI (blue). (B) Enumeration of stained cells indicated that *Fut2*^*+/+*^ mice have higher numbers of CD68^+^ cells in colon mucosa at day 7 p.i. (C) MPO signal (red) showed increased recruitment of neutrophils in *Fut2*^*+/+*^ mice. UEA-I (green) positive staining was detected only in *Fut2*^*+/+*^ mice. Scale bars 50 μm. (D) Flow cytometry revealed a higher frequency of CD4^+^ (CD3^+^CD4^+^) cells present in colonic lamina propria of *Fut2*-expressing mice compared to *Fut2-*deficient mice (n = 4–6). Scale bars, 50 μm. *p<0.05, Mann-Whitney test.

Next, colon tissue sections were analyzed by immunohistochemical staining and subsequent quantification of CD68- and MPO-positive cells, which represent macrophages and neutrophils, respectively. Consistent with elevated histopathological scores, significantly higher numbers of recruited neutrophils and macrophages were detected in the colon tissue of *Fut2*^+/+^ mice compared to *Fut2*^-/-^ mice (Fig 2A–2C). In addition, a significantly stronger infiltration of CD4^+^ T lymphocytes in the colonic lamina propria of *Fut2*^+/+^ mice compared to *Fut2*^-/-^ mice was detected by immunofluorescence staining and by flow cytometry (Fig 2A and 2D). No statistically significant differences were found in the numbers or infiltrate composition with respect to cytotoxic T lymphocytes, B lymphocytes, or dendritic cells by flow cytometric quantification of CD8^+^, CD19^+^, and CD11c^hi^ cells, respectively (S3 Fig).

To summarize, *Fut2*-expressing mice exhibited higher bacterial load in the intestine at later time points, which was also associated with an increase in inflammation assessed by histopathology and lipocalin-2 levels, demonstrating that *Fut2-*mediated fucosylation in the intestine plays an important role in *Salmonella*-triggered inflammation and colonization of the intestine.

### Std fimbriae mediate adhesion to intestinal epithelial cells in a fucose-dependent manner

**Fig 3:**
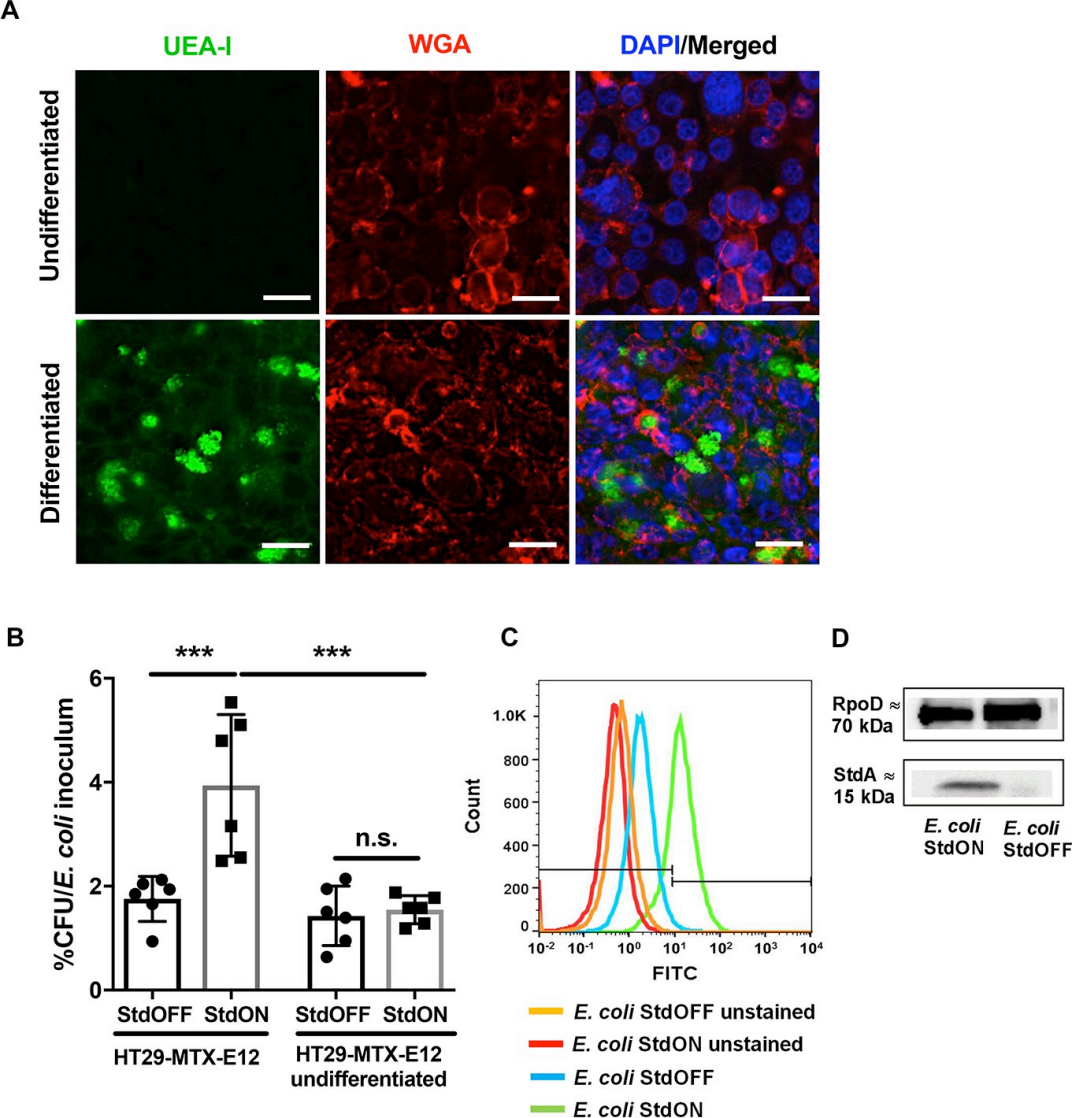
Std fimbriae mediate adhesion to intestinal epithelial cell culture in a fucose-dependent manner. (A) UEA-I (green) and WGA (red) staining in formalin-fixed HT29-MTX-E12 cells, at day 1 after seeding (undifferentiated) and at day 21 after seeding (differentiated). Positive UEA-I staining was present only in differentiated cells. Scale bars, 20 μm. (B) *E*. *coli* overexpressing *stdABCD* operon (StdON) displayed higher adherence to differentiated HT29-MTX-E12 cells; this effect was absent in differentiated HT29-MTX-E12 cells infected with *E*. *coli* StdOFF and in undifferentiated HT29-MTX-E12 cells infected with either strain. ***p<0.001; n.s. = not significant, ANOVA with Tukey’s multiple comparison test. (C) Flow cytometry analysis of *std* expression in *E*. *coli* strains. (D) Specificity of the serum and Std expression were additionally confirmed by Western blotting. *E*. *coli* RpoD (70kDa) was used as a control.

The Fut2 protein facilitates intestinal epithelial fucosylation by catalyzing the addition of L-fucose residues via an α(1,2) linkage to the terminal β-D-galactose residue of glycans. The *std* operon of *S*. Typhimurium encodes a fimbrial adhesin known to be important for the attachment to fucosylated structures on intestinal epithelial cells [16]. To investigate the role of fucose-Std fimbriae interaction during *Salmonella* adherence, human intestinal epithelial cell lines HT29-MTX-E12 and Caco-2 were utilized. HT29-MTX-E12 are colon epithelial cells that differentiate into goblet-like cells and produce mucus after three weeks of *in vitro* culture [19]. *Ulex europaeus* agglutinin I (UEA-I) lectin staining revealed extensive fucosylation of cell surface and mucus in the differentiated HT29-MTX-E12 cells in contrast to undifferentiated HT29-MTX-E12 cells. Wheat germ agglutinin (WGA) lectin staining for the ubiquitously expressed N-acetylglucosamine was positive in both differentiated and undifferentiated HT29-MTX-E12 cells (Fig 3A).

Previous studies have shown that bacterial expression of Std fimbriae is a subject of complex and tight regulation, both *in vivo* and *in vitro* [20,21]. Only a very small proportion of the *Salmonella* population express *std* fimbriae *in vitro* [22] and the *std* operon is completely absent in *E*. *coli*. Therefore, to analyze the role of Std fimbriae *in vitro*, an inducible expression plasmid containing the *Salmonella stdABCD* operon encoding the structural genes of Std fimbriae was transformed into a common laboratory *E*. *coli* K-12 and afimbriated *E*. *coli* ORN172. Upon induction with anhydrotetracycline, Std fimbriae were expressed by *E*. *coli* (referred as *E*. *coli* StdON) as confirmed by flow cytometry and Western blotting (Fig 3C and 3D). HT29-MTX-E12 cells were infected with either *std*-expressing (*E*. *coli* StdON) or non-expressing bacteria (*E*. *coli* StdOFF). In contrast to *E*. *coli* StdOFF, only *E*. *coli* StdON strain showed increased adherence to differentiated HT29-MTX-E12 cells (Fig 3B). Importantly, expression of Std fimbriae had no effect on adhesion of *E*. *coli* StdON to undifferentiated HT29-MTX-E12 cells which do not contain fucosylated glycoproteins (Fig 3B). Similarly, *E*. *coli* ORN172 StdON bacteria adhered significantly better to Caco-2 cells compared to *E*. *coli* lacking Std expression. Addition of fucose-binding UEA-1 lectin to the cells prior to infection abrogated the adhesion of the *std*-expressing strain. In contrast, addition of dolichus biflorus aggluttinin (DBA), which binds to N-acetylgalactosamine, did not affect binding of *E*. *coli* ORN172 StdON to Caco-2 cells (S4A and S4B Fig). Atomic force microscopy showed Std piliation of *E*. *coli* ORN172 StdON and the absence of pili in the empty vector control bacteria (S4C Fig). In conclusion, Std fimbriae are important for binding α(1,2)-fucosylated residues on cell lines corroborating the results by Chessa and colleagues [16].

**Fig 4:**
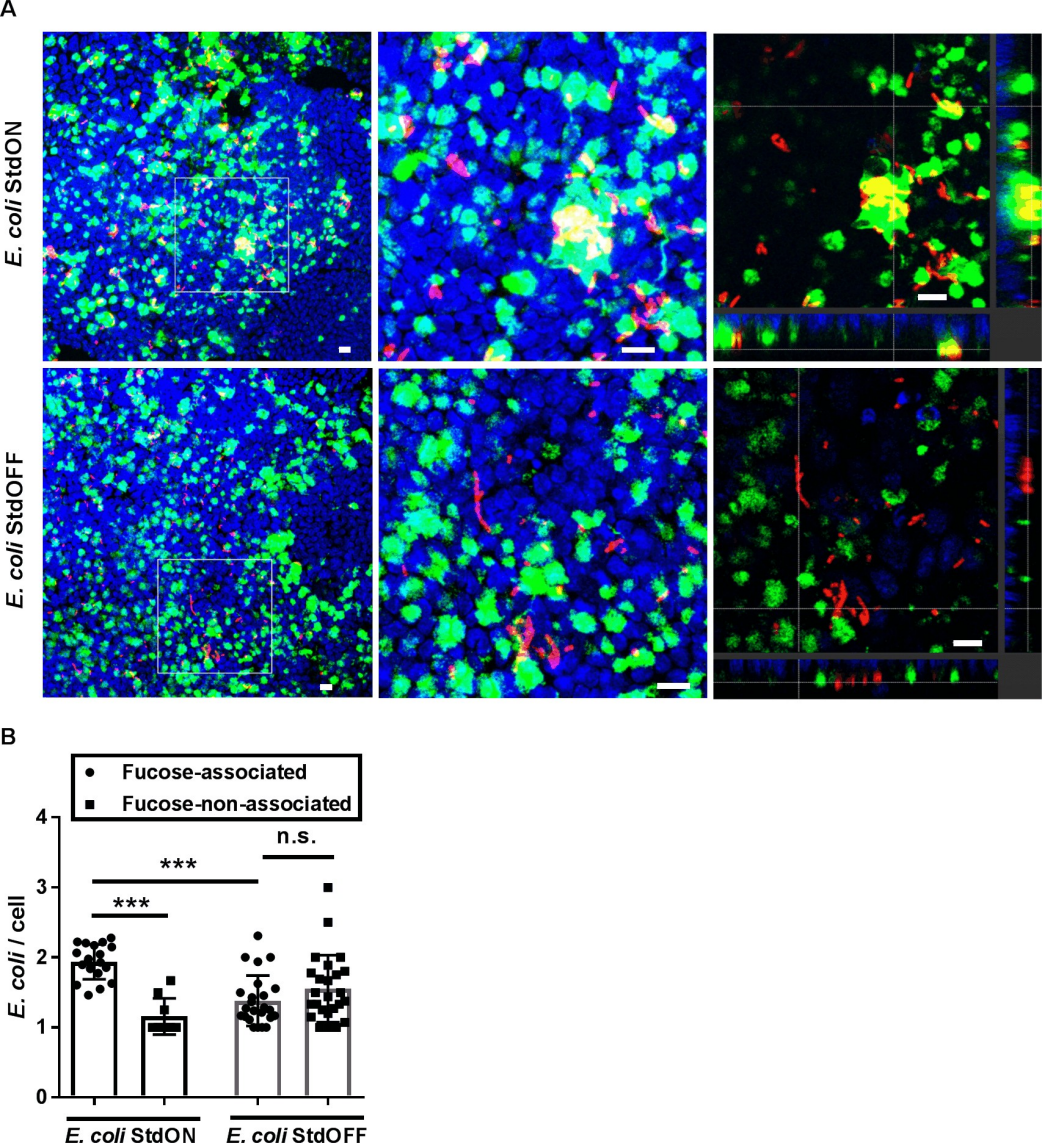
*E*. *coli* overexpressing Std fimbriae bind to fucosylated cells in primary intestinal epithelial monolayers. (A) 2D monolayers of *Fut2*^+/+^ murine colon organoids were infected with either *E*. *coli* StdON or *E*. *coli* StdOFF and immunofluorescently stained using anti-*E*. *coli* and UEA-1 lectin. Image analysis of the infected monolayers revealed co-localization of Std-expressing *E*. *coli* bacteria (red) and the α(1,2)-fucosylated cells (green) (A, enlarged region and its orthogonal section). Nuclei were stained with DAPI (blue). Scale bar, 20 μm. (B) Fucose-associated and non-associated *E*. *coli* were quantified microscopically using at least 20 fields of view (FOV) per sample. A significantly higher numbers of the fucose-associated bacteria were detected when Std fimbriae were expressed. Data represents four independent biological repetitions. ***p<0.001; n.s. = not significant, ANOVA with Tukey’s multiple comparison test.

Next, Std-dependent bacterial adherence to primary epithelial cells was investigated. To this end, primary intestinal epithelial cells were isolated from *Fut2*^+/+^ mice and cultivated as three-dimensional organoids in matrigel. These enteroids were expanded and seeded onto transwell filters resulting in the formation of a 2D monolayer consisting of various primary epithelial cell types. Monolayer barrier integrity and the degree of differentiation were evaluated by measuring transepithelial electrical resistance. Polarized monolayers were infected with *E*. *coli* StdON and StdOFF bacteria and adherence was analyzed by immunofluorescence. We counted the number of *E*. *coli* bacteria attached to UEA-1-positive and -negative cells. *E*. *coli* StdON bacteria were primarily associated with fucosylated cells, while *E*. *coli* StdOFF adhered equally to fucosylated and non-fucosylated cells (Fig 4A). Furthermore, a significantly higher number of the α(1,2)-fucose-associated *E*. *coli* StdON cells compared to the α(1,2)-fucose-associated *E*. *coli* StdOFF bacteria was detected (Fig 4B). Overall, this data demonstrate that *std-*expressing bacteria preferentially bind to fucosylated cells.

### Std fimbriae-fucose interaction is critical for *Salmonella*-induced inflammation and colonization

Using cut sections of the cecum of CBA/J mice, it was previously demonstrated that purified Std fimbriae of *S*. Typhimurium are able to bind terminal α(1,2)-fucose residues in the mucosa [16]. However, the functional consequences of this interaction for disease development, as well as the extent of Std fimbriae expression *in vivo* are not known.

**Fig 5:**
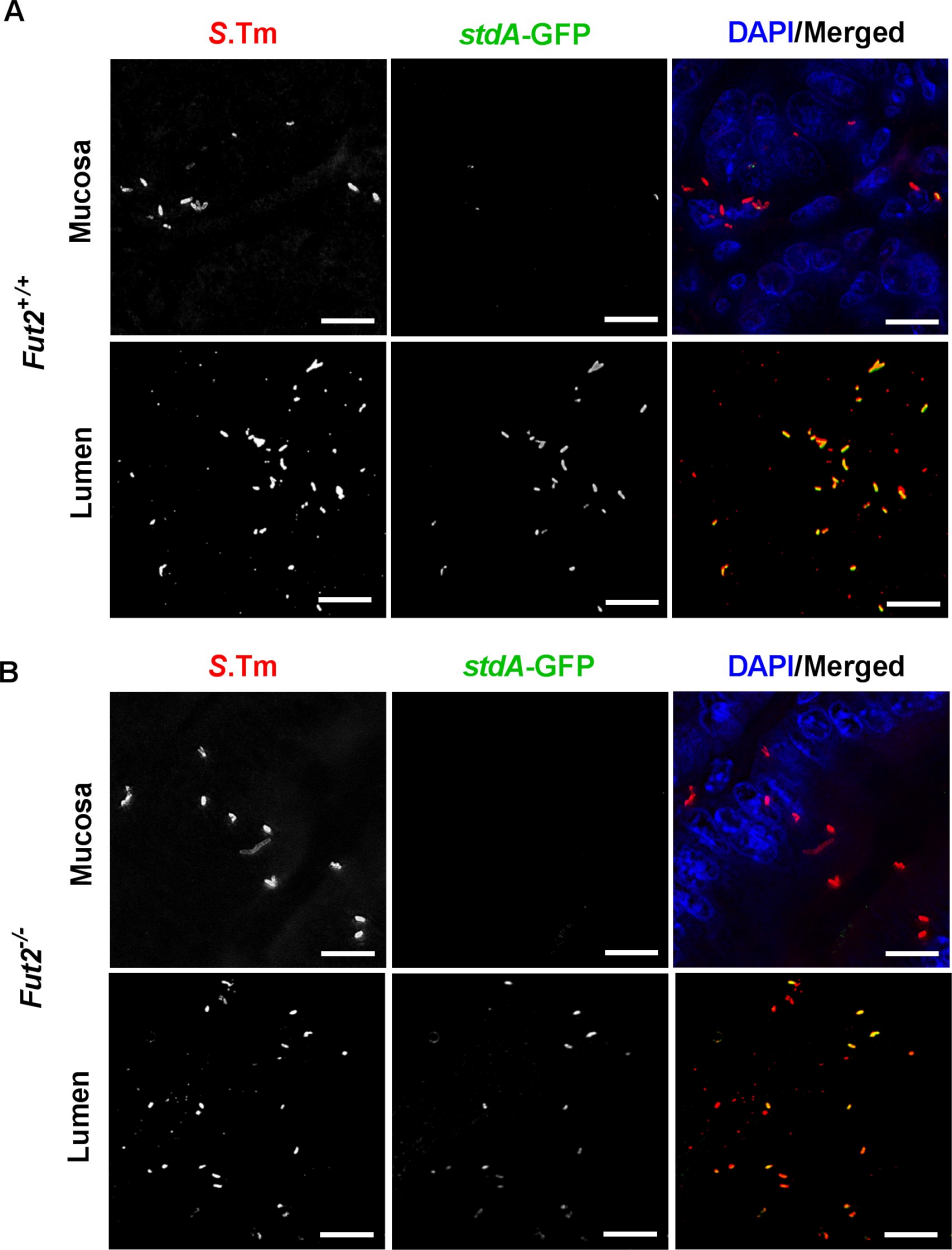
Std fimbriae are differentially expressed *in vivo*. *Fut2*^+/+^ (A) and *Fut2*^*-/-*^ (B) mice (n = 4) were infected with the reporter *S*. Typhimurium Δ*aroA stdA*stop::*gfp* strain. Mice were sacrificed at day 7 p.i. and colon sections were subjected to immunohistochemistry. GFP-positive bacteria were detected with an anti-GFP antibody. Std(GFP)-expressing *S*. Typhimurium were localized in the lumen of the colon, but not within the mucosa. Scale bars, 10 μm.

To assess Std fimbriae production *in vivo*, *stdA* gene expression was first examined using RT-qPCR. Similar levels of *stdA* gene expression were detected in the colon of both *Fut2*^+/+^ and *Fut2*^-/-^ mice infected with *S*. Typhimurium Δ*aroA* strain (S5A Fig). In order to determine whether the absence of *std* or the presence of intestinal fucosylated glycans affects expression of fucose or 1,2-propanediol utilization pathways we quantified levels of *fucI* and *pduBC* by RT-qPCR. We saw comparable levels of these genes expressed in either mouse strain in *std*-containing and *std*-deficient bacteria (S5B and S5C Fig). In order to look more closely at the spatial regulation of Std expression, we used reporter strains of *S*. Typhimurium containing a *stdA*stop::*gfp* fusion [22] and staining of tissue sections with anti-Std serum. Std was observed to be specifically expressed in the lumen of the large intestine of both *Fut2*^+/+^ and *Fut2*^-/-^ mice on day 1 (S6 Fig) and day 7 p.i. (Fig 5 and S7 Fig). In contrast, Std-expressing *Salmonella* were not observed after invasion of the mucosa (Fig 5, S6 and S7 Figs). These data demonstrate that there is a tight spatial regulation of Std-expression whereby Std fimbriae are expressed prior to invasion of the large intestine.

**Fig 6:**
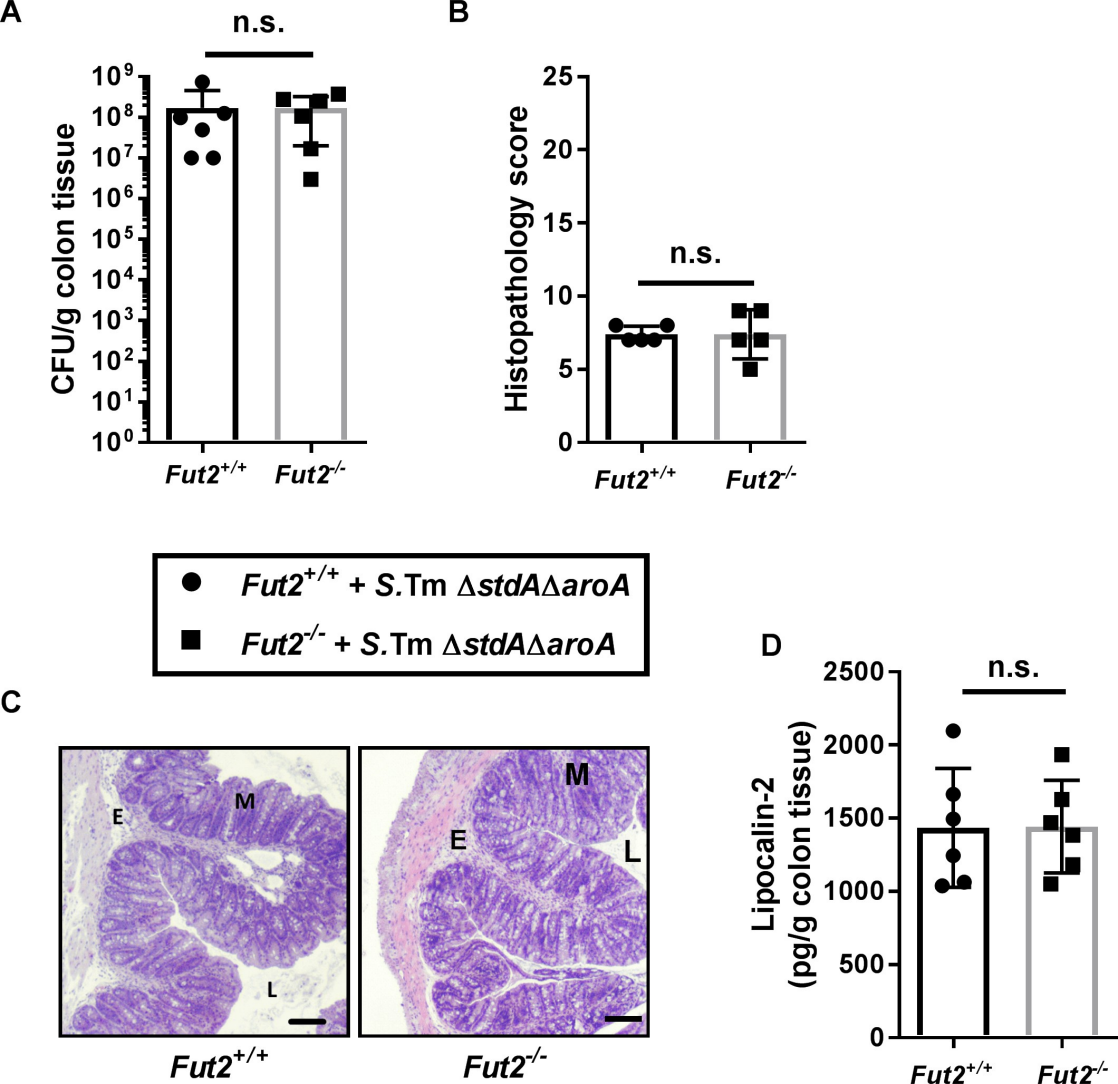
Std fimbriae-fucose interaction impacts *Salmonella*-induced inflammation and colonization of the colon. (A) *S*. Typhimurium Δ*aroA*Δ*stdA* loads were determined in colon by plating homogenates on LB agar with streptomycin. Both *Fut2*^*+/+*^ and *Fut2*^*-/-*^ mice showed similar bacterial loads. (B) Histology scoring revealed comparable inflammation in *Fut2*^*+/+*^ and *Fut2*^*-/-*^ infected colons at day 7 p.i. (C) H&E staining of colon tissue sections at 7 days p.i. Scale bars, 50 μm. Moderate numbers of necrotic epithelial cells, mild inflammatory cell infiltration within intestinal mucosa, and mild submucosal edema were observed in both *Fut2*^*+/+*^ and *Fut2*^*-/-*^ mice upon *S*. Typhimurium Δ*aroA*Δ*stdA* infection. (D) Lipocalin-2 levels were measured by ELISA in supernatants of colon tissue homogenates. Graph shows representative data of three independent experiments (n = 5–6 per group). n.s = not significant, Mann-Whitney test.

*Fut2*^-/-^ mice lack terminal fucose on intestinal epithelium [23]. To test whether epithelial fucosylation directly affects *Salmonella* colonization via interaction with Std fimbriae, the *stdA* mutation was transferred into the *S*. Typhimurium Δ*aroA* background strain. This mutant strain lacked functional Std fimbriae (*S*. Typhimurium Δ*aroA*Δ*stdA*) yet had the same growth rate and motility as the parental *S*. Typhimurium Δ*aroA* strain (S8 Fig). *Fut2*^+/+^ and *Fut2*^-/-^ mice were then infected with *S*. Typhimurium Δ*aroA*Δ*stdA* via oral gavage for 7 days. In contrast to *S*. Typhimurium Δ*aroA* (Fig 1), the *S*. Typhimurium Δ*aroA* Δ*stdA* strain colonized the colon of *Fut2*^+/+^ and *Fut2*^-/-^ mice to similar levels (Fig 6A). In addition, histopathology scores showed similar intestinal inflammation of *Fut2*^+/+^ and *Fut2*^-/-^ mice. H&E staining of colonic tissue showed moderate numbers of necrotic epithelial cells, mild inflammatory cell infiltration within intestinal mucosa and mild submucosal edema (Fig 6B and 6C), which was similar in both mouse genotypes. In addition, lipocalin-2 concentrations were similar in colons of both *Fut2*^+/+^ and *Fut2*^-/-^ mice after infection with *S*. Typhimurium Δ*aro*A Δ*std*A (Fig 6D).

**Fig 7:**
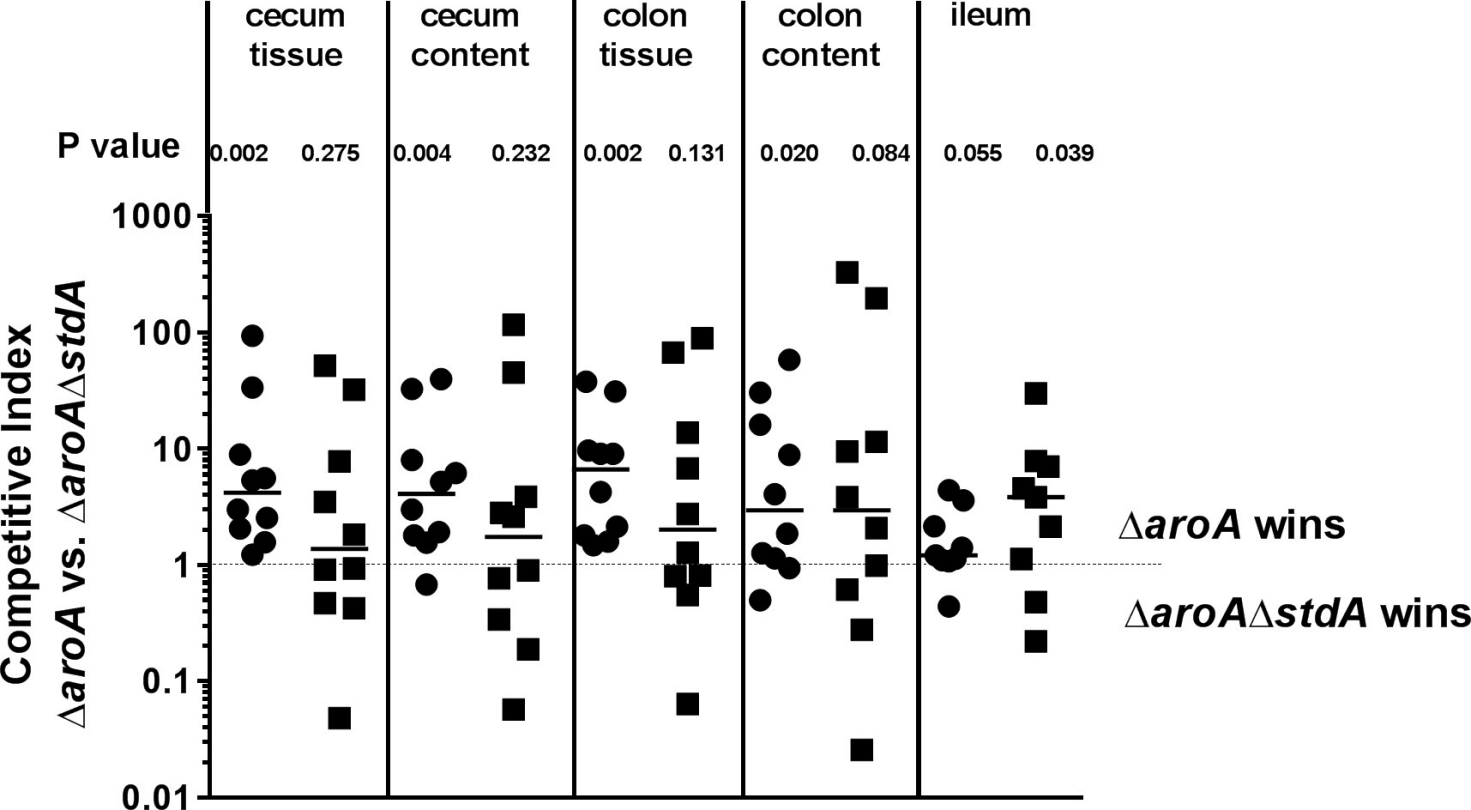
Std fimbriae provides a competitive advantage in a fucosylated environment. Competitive index (CI) was determined by infecting *Fut2*^*+/+*^ and *Fut2*^*-/-*^ mice (n = 10 per group) with an equal amount of *S*. Typhimurium Δ*aroA* and *S*. Typhimurium Δ*aroA*Δ*stdA*. Organ and feces homogenates were plated on LB agar containing streptomycin (total *Salmonella*) and on LB plates with streptomycin+kanamycin (*S*. Typhimurium Δ*aroA*Δ*stdA* only). (A) In cecum and colon, *S*. Typhimurium *ΔaroA* outcompeted the isogenic *ΔstdA* mutant in *Fut2*^*+/+*^ mice, but not in *Fut2*^*-/-*^ mice. Graph shows data for individual mice and median values, p values are indicated for each group as determined by Wilcoxon signed-rank test.

In order to further explore whether Std fimbriae play a role in the colonization of *S*. Typhimurium in the presence or absence of host intestinal fucosylation, competitive index (CI) experiments were performed by orogastrically infecting both *Fut2*^+/+^ and *Fut2*^-/-^ mice with equal numbers of *S*. Typhimurium Δ*aroA* and *S*. Typhimurium Δ*aroA* Δ*stdA*. Fecal pellets were collected at 1, 3, and 5 days p.i. and bacterial counts of both strains were determined. After 7 days, the bacterial load in intestinal tissues and luminal content was determined and the CI ratio of the two strains was calculated. Interestingly, in cecum and colon, *S*. Typhimurium Δ*aroA* significantly outcompeted the isogenic Std-deficient strain in *Fut2*^+/+^ mice, but not in *Fut2*^-/-^ mice (Fig 7). Accordingly, CI results from feces at day 1, 3, and 5 showed that *S*. Typhimurium Δ*aroA* outcompeted the isogenic Δ*stdA* mutant in *Fut2*^*+/+*^ mice (S9 Fig). Taken together, our *in vivo* data demonstrate that Std fimbriae are important for *Salmonella* colonization, persistence, and induction of inflammation in a fucosylated host environment.

## Discussion

Variation in human glycosylation influences various metabolic diseases, cancers, inflammatory diseases, and susceptibility to infectious pathogens. Genome-wide association studies show that *FUT2* nonsense polymorphisms are associated with increased risk for Crohn’s disease [10] and primary sclerosing cholangitis [24]. Genetic variation in *FUT2* is also linked to susceptibility to infections with bacterial and viral pathogens including *Helicobacter pylori* [25], norovirus [26, 27], Enterotoxigenic *E*. *coli* [5], and progression of HIV [28]. In this study, we investigated the role of *Fut2* expression for *S*. Typhimurium infection and found that Std fimbriae-fucose interaction was important for *Salmonella*-induced inflammation and colonization.

Host mucosal glycans can influence the susceptibility to infection indirectly or directly. Indirectly, glycan-dependent differences in microbiota composition may contribute to the susceptibility to infection with a particular pathogen. For example, we previously reported an influence of the histo-blood group related glycosyltransferase gene *B4galnt2* on host susceptibility to *S*. Typhimurium infection. The expression of B4galnt2 in the gut results in differences in microbial composition which in turn affect the extent of *Salmonella* colonization, and hence, disease pathology [29]. Many complex carbohydrates degraded by the intestinal microbiota produce metabolites that can be utilized by *Salmonella* Typhimurium and *Clostridium difficile* thereby facilitating their expansion within the gut [4]. Host glycans can also directly influence a host’s susceptibility to infection by modulating bacterial attachment to host tissues. Many bacteria produce specific adhesins which bind to host glycans. For example, the *H*. *pylori* adhesin BabA mediates adherence to the gastric mucosa of individuals with fucosylated ABO(H)/Lewis b blood group antigens [30, 31]. Norovirus (strain GII.4) and rotavirus (strains with spike protein VP8) encode adhesins which bind α(1,2)-fucosylated glycans resulting in increased susceptibility in individuals with a secretor phenotype [32,33].

While *Fut2* expression in the small intestine is inducible by ILC3-derived IL-22 [17], the large intestine is constitutively fucosylated [8]. Mice lacking *Fut2* are more susceptible to *S*. Typhimurium infection at an early time point post infection, as demonstrated by Goto and colleagues [17] and confirmed in the present study. In contrast, we demonstrate that at later time points, a lack of *Fut2* expression is associated with decreased intestinal colonization, pathology, and inflammatory responses. It has been hypothesized that at one day post *S*. Typhimurium infection, α(1,2)-fucose-containing glycans secreted from goblet cells may interfere with the attachment of *Salmonella* to intestinal epithelial cells, although this has not been proven so far [34]. Our study suggests that at later time points post infection, *Salmonella* exploits α(1,2)-fucose-containing glycans present in the intestine of *Fut2*^+/+^ mice to their advantage. Fucose and its metabolic products such as 1,2-propanediol can be utilized by *S*. Typhimurium as carbon and energy sources [35]. *In vivo*, during intestinal inflammation, 1,2-propanediol is generated and serves as a nutrient source for *Salmonella* [36]. Our data do not show any differences in the expression of *fucI* and *pduBC* in *Fut2*^+/+^ and *Fut2*^-/-^ mice, even in the absence of *stdABCD* genes, suggesting that these pathways are not regulated by Std fimbriae.

It was previously shown that purified Std fimbriae of *S*. Typhimurium can bind terminal α(1,2)-fucose residues [16]. *Salmonella enterica* encodes up to 13 fimbrial and at least 7 non-fimbrial adhesins depending on the serovar [13,37]. The production of adhesins involves complex and tight regulation since inappropriate expression could be detrimental for bacterial colonization and pathogenesis. While the majority of adhesins are expressed in temporal and spatially highly controlled manner during animal infections, they are often not produced under laboratory conditions [20, 38]. *In vitro* expression of the *S*. Typhimurium *std* operon is bistable resulting in the emergence of a minor subpopulation of Std-positive cells [22]. Therefore, to investigate the role of Std fimbriae *in vitro* we took advantage of an Std-expressing *E*. *coli* strain. Our *in vitro* infection experiments revealed enhanced attachment of Std-expressing *E*. *coli* to fucosylated cell lines, which could be specifically blocked by the addition of the α(1,2)-fucose-binding lectin UEA-I. These observations confirm the findings of Chessa and colleagues [16] and were further corroborated by preferential binding of Std-expressing *E*. *coli* to fucosylated cells in primary epithelial monolayers. Altogether, bacteria expressing Std fimbriae exhibited increased adhesion to human cell lines and murine intestinal crypt organoids when terminal α(1,2)-fucose was present.

The extent and localization of Std fimbriae expression *in vivo* was not previously known. Notably, we observed the spatial expression pattern of Std fimbriae *in vivo*. In this work, we present, for the first time, evidence that Std fimbriae are differentially produced during intestinal infection. Std-positive *Salmonella* were found predominantly in the intestinal lumen, which corroborates the existence of a regulatory fimbrial switch.

Previous work has shown that Std fimbriae are required for the long-term *S*. Typhimurium colonization of CBA mice [39]. In agreement with these data, we show that Std fimbriae are important for persistence in C57Bl/6 mice and demonstrate that this is strictly dependent on the ability of Std fimbriae to adhere to fucosylated host glycoproteins or glycolipids in the large intestine. While the expression of α(1,2)-fucose on enterocytes and goblet cells is facilitated by Fut2, M cells in the follicle associated epithelium overlying Peyer’s patches [40] and Paneth cells [41] are α(1,2)-fucosylated via Fut1. Thus, based on our CI data, we can conclude that *Fut2*-dependent fucosylation is important for *Salmonella* persistence only in the large intestine. Furthermore, we present evidence that Std fimbriae are also important for the induction of host intestinal pathology and inflammation in a Fut2-dependent manner.

In conclusion, our results demonstrate a substantial role for glycosylation of the intestinal mucosa in the susceptibility to *S*. Typhimurium infection. Std fimbriae binding of terminal α-1,2 fucose residues mediate bacterial adherence to host glycoproteins or glycolipids. Taken together, our results conclusively demonstrate that host fucosylation in the intestine is exploited by *S*. Typhimurium during the course of infection in a mechanism that requires *Salmonella* expression of Std fimbriae.

## Material and methods

### Mice

B6.129X1-Fut2^tm1Sdo^/J (*Fut2*^-/-^) [42] mice were purchased from the Jackson Laboratory and intercrossed with wild-type C57BL/6J (*Fut2*^+/+^) mice. Mice were backcrossed for 14 generations. Heterozygous breeding pairs produced litters of mixed genotypes. *Slc11a1* (Nramp1) is an important resistance gene for *S*. Typhimurium. 129X1 mice harbor *Slc11a1* resistant alleles while in C57Bl/6J mice a point mutation results in *Slc11a1* sensitive alleles. The *Slc11a1* genotype of mice was verified as described [[43]] using a common reverse primer and forward primers (S2 Table) specific for the sensitive and resistant allele, respectively. All mice were homozygous for the *Slc11a1* sensitive allele. Mice were housed together under specific pathogen-free conditions in individually ventilated cages (IVC). Standard chow and water were provided *ad libitum*. Experiments were conducted in the animal facilities of the University of Kiel and Hannover Medical School in Germany.

### Ethics statement

Animal experiments were conducted in direct accordance with the German Animal Protection Law consistent with the ethical requirements and approval of the Animal Care Committee of the Ministry of Energy, Agriculture, the Environment and Rural Areas of Schleswig-Holstein, Germany (protocol # V244-7224.121.3 (99-10/10)) and by the Niedersächsisches Landesamt für Verbraucherschutz und Lebensmittelsicherheit (protocol # 33.12-42502-04-16/2071).

### Bacteria

*S*. Typhimurium SL1344 (*S*. Tm) [44], *S*. Tm Δ*aroA* [18] were grown at 37°C with shaking in lysogeny broth (LB) supplemented with 100 μg/ml streptomycin. *S*. Tm Δ*aroA*Δ*stdAB* (referred to as *S*. Tm Δ*aroA*Δ*stdA*) double mutant was generated by P22 phage transduction of the Δ*stdAB* deletion from the *S*. Tm NCTC 12023 Δ*stdAB* [38] to the *S*. Tm SL1344 Δ*aroA* background. *S*. Tm Δ*aroA stdA*stop::*gfp* strain was created by P22 transduction of the Δ*aroA* deletion into the parental *S*. Tm *stdA*stop::*gfp* strain [22]. For *in vivo* infection, *S*. Tm Δ*aroA*Δ*stdA* and *S*. Tm Δ*aroA stdA*stop::GFP strains were grown overnight in LB broth supplemented with kanamycin 50 μg/ml at 37°C. The *E*. *coli* Std strain containing the anhydrotetracyclin (AHT)-inducible *Salmonella stdABCD* operon was generated by electroporation of the plasmid p4394 [45] into *E*. *coli* Turbo (New England Biolabs) K-12 strain (referred as WT). For *in vitro* infection experiments, *E*. *coli stdABCD* was grown overnight in LB broth supplemented with carbenicillin 50 μg/ml at 37°C and then grown to logarithmic phase in the presence (StdON) or absence (StdOFF) of AHT (IBA, 100 ng/ml). The non-fimbriated *E*. *coli* ORN172 strain [38] harboring the empty vector or a plasmid for expression of the *Salmonella stdABCD* operon under control of the *tetR P*_*tetA*_ expression cassette were grown in LB broth. Expression was induced by addition of 100 ng/ml anhydrotetracycline (AHT). Imaging of bacteria by atomic force microscopy (AFM) was performed as previously described [45].

### *S*. Typhimurium infection of mice

*Fut2*^-/-^ and wild-type (*Fut2*^+/+^) littermates were pretreated by oral gavage with 20 mg of streptomycin (Sigma-Aldrich) 24 hours before infection. Mice were orally gavaged with either 3x10^6^ *S*. Tm, *S*. Tm Δ*aroA*, *S*. Tm Δ*aroA*Δ*stdA*, *S*. Tm *stdA*stop::*gfp*, or *S*. Tm Δ*aroA stdA*stop::*gfp* in 100 μl HEPES buffer (100 mM, pH 8.0; PAA Laboratories). Control mice (mock infection) were given 100 μl HEPES buffer. To enumerate luminal and tissue-invaded bacteria, colon and cecum tissues were harvested and the intestinal contents were separated from the tissues. Tissues were then treated with PBS containing 100 μg/ml gentamicin at 4°C for 30 minutes to kill bacteria on the tissue surface. Intestinal tissues and intestinal contents were homogenized, serially diluted, and plated on LB agar containing streptomycin (100 μg/ml).

### Competitive index (CI)

*Fut2*^+/+^ and *Fut2*^-/-^ mice were pre-treated with 20 mg of streptomycin and infected with a mixture of *S*. Tm Δ*aroA* and *S*. Tm Δ*aroA*Δ*stdA* strains (1:1, 3x10^6^ total bacteria per mice). During the infection, fecal pellets were collected at days 1, 3, and 5 p.i. At 7 days p.i., mice were sacrificed and bacterial loads in cecum tissue, cecum content, colon tissue, colon content, and ileum were enumerated. Feces and intestinal tissues were homogenized and plated on selective LB agar plates supplemented with either streptomycin (100 μg/ml) alone (to determine total *Salmonella* load) or with streptomycin (100 μg/ml) and kanamycin (50 μg/ml) (to enumerate *S*. Tm Δ*aroA*Δ*stdA*). The number of *S*. Tm Δ*aroA* was calculated by subtracting the CFU counts of *S*. Tm Δ*aroA*Δ*stdA* from the total *Salmonella* counts. Competitive index (CI) was calculated as the ratio of (Δ*aroA* / Δ*aroA*Δ*stdA*) at the time of sampling divided by (Δ*aroA* / Δ*aroA*Δ*stdA*) of the inoculum.

### Motility assay

Motility was assessed by inoculating motility agar plates (10 g/l tryptone, 5 g/l NaCl, and 0.3% Bacto-agar) with saturated bacterial cultures grown overnight in LB broth. Motility halos were compared after incubation at 37°C for 6 hours.

### Pathology and histology

Organs were fixed in 10% formalin, dehydrated with ethanol, and embedded in paraffin. Paraffin sections were stained with hematoxylin-eosin (H&E) according to standard laboratory procedures. Histological scores of ceca and colons were determined as previously described [46]. Briefly, pathological changes were assessed by evaluating the presence of necrotic epithelial cells and neutrophils in lumen; desquamation and ulceration in surface epithelial cells; crypt abscesses; infiltrating inflammatory and immune cells in mucosa and submucosa layer; and the formation of edema in submucosa layer.

### *In vitro* infection

Human colon epithelial clonal cell line, HT29-MTX-E12 [47,48] (a kind gift from Marguerite Clyne, University College Dublin), and the colorectal carcinoma cell line, Caco-2BBe1 (ATCC CRL-2102), were grown in DMEM supplemented with 10% fetal bovine serum (Biochrom) and 1% MEM non-essential amino acids solution (Gibco, Life Technologies). Cells were seeded in 24 well plates and incubated at 37°C in a humidified 5% CO_2_ atmosphere. Cells were grown for 7 days (Caco-2-BBe1) or 21 days (HT29-MTX-E12) to achieve differentiation of the monolayers. Cells were then infected with *E*. *coli* StdOFF or *E*. *coli* StdON, *E*. *coli* ORN172 StdOFF or *E*. *coli* ORN172 StdON for 30 min at an MOI of 50. For quantification of adherence, cells were washed four times with PBS and lysed in PBS containing 1% (v/v) Triton X-100. The number of adherent bacteria was determined by serial dilutions plating. Where indicated, 30 min prior to infection, cells were incubated with medium in presence of 0.3 mM UEA-I or DBA lectins (CosmoBio) at 37°C in a humidified 5% CO_2_ atmosphere.

### Intestinal epithelial organoids

Primary colonic and ileal crypts were isolated from *Fut2*^+/+^ mice as described [49] with modifications. Briefly, mice were sacrificed by cervical dislocation. Intestines were opened longitudinally, cut into small pieces and washed three times with 5 ml of ice-cold DPBS. Tissues were then incubated in 10 ml of ice-cold crypt chelating buffer (10 mM EDTA in DPBS) for 90 min on an orbital shaker. The supernatant was discarded and the settled tissue fragments were resuspended twice in 5 ml ice-cold DPBS. Crypts were centrifuged for 5 min at 800 rpm at 4°C and pellets were resuspended in 1 ml ice-cold DPBS. About 100 crypts were resuspended in 25 μl organoid medium (Advanced DMEM/F12 medium (Thermo Fischer Scientific) supplemented with 2 mM GlutaMax, 50% L-WRN-Supernatant (ATCC CRL3276), 10 mM HEPES, 100 U/ml penicillin, 100 μg/ml streptomycin, B27 supplement, 50 ng/ml recombinant mouse epidermal growth factor (rm EGF), 500 nM A83-01 (Tocris), 10 μM SB202190 (Tocris), 10 nM Gastrin I (Tocris), 1 mM N-Acetyl-L-cysteine (Sigma), and 10 μM Y27623 (Tocris)). 25μl Matrigel (Corning) was added into a well of a pre-warmed 24-well plate. The plate was incubated for 0.5 h in a 37°C incubator with 5% CO2 to allow complete polymerization of the Matrigel. Crypts were covered with 500 μl of the organoid medium. To form 2D monolayers, 3D organoids were resuspended in ice-cold PBS and centrifuged at 1500 rpm for 10 min at 4°C. Pellets were resuspended in 1 ml warm 0.05% trypsin/EDTA and incubated for 5 min at 37°C in a water bath. Organoids were dissociated by pipetting and washed with ice-cold DMEM/10% FCS and resuspended in monolayer medium (Advanced DMEM/F-12, 50% L-WRN-Supernatant, 20% fetal bovine serum, 2 mM L-glutamine, 100 U/ml penicillin, 0.1 mg/ml streptomycin, 10 μM Y-27632, 50 ng/ml rm-EGF). Cell suspensions were seeded onto Transwell permeable supports (polyester; 6.5 mm diameter; 0.4 μm pore size; Corning) that had been coated for 2 h at 37°C with Matrigel (diluted 1:40 in PBS). Monolayer medium was replaced every two days and monolayer barrier integrity was evaluated by measuring transepithelial electrical resistance (TEER) using a volt-ohmmeter (Millipore). On day 5 after seeding, medium was changed to differentiation medium (Advanced DMEM/F-12, 5% L-WRN-Supernatant, 20% fetal bovine serum, 2 mM L-glutamine, 50 ng/ml rm-EGF, 5 μM DAPT). For the next two days, differentiation medium was changed every day and TEER was measured. 2D monolayers were infected with either *E*. *coli* StdON or *E*. *coli* StdOFF (7 x 10^7^ bacteria per Transwell), incubated for 30 min at 37°C, washed four times with PBS, and fixed with 4% paraformaldehyde (PFA). Adherent fucose-associated and non-associated bacteria were counted microscopically, using at least 20 fields of view (FOV) per sample.

### Immunofluorescence

Formalin-fixed paraffin-embedded tissue sections (5 μm) were deparaffinized and rehydrated. Heat-induced epitope retrieval was performed using 10 mM sodium citrate buffer (pH 6.0) and blocking was achieved using 2% normal goat serum (NGS). The following antibodies were used for immunohistochemistry (see S1 Table for a full description): anti-StdA serum [38], *Salmonella* O Antiserum Group B (BD Difco), anti-GFP (DSHB), CD3 (Abcam), CD68 (Abcam), myeloperoxidase (MPO) (Thermo Fisher Scientific), and fluorescently-labeled secondary antibodies (Invitrogen). Counterstaining of nuclei was done with 4,6-Diamidin-2-phenylindol (DAPI) (Invitrogen). HT29-MTX-E12 and Caco-2 Bbe1 cells were seeded on coverslips in 24 well plates and fixed with 4% PFA before and after differentiation. Blocking of non-specific binding was done using 2% NGS. Fluorescently-labeled lectins UEA-1 (*Ulex Europaeus* agglutinin-1) (CosmoBio) and WGA (wheat germ agglutinin) (Vector laboratories) were used to visualize α(1,2)-fucosylation and the presence of sialic acid / N-acetylglucosaminyl residues, respectively. Fixed primary epithelial cell monolayers were stained with the anti-*E*.*coli* antibody (Abcam), UEA-1 lectin (CosmoBio), and DAPI. Images were obtained on a Zeiss Apotome.2 microscope using AxioVision 4.9.1 software (Zeiss) and on a Leica DMi8 confocal laser scanning microscope using LAS X 3.3.0.16799 software (Leica). Brightness and contrast were adjusted using ImageJ 1.52e software.

### Flow cytometry

Isolation of colonic lamina propria cells was achieved using the Lamina Propria Dissociation Kit (Miltenyi Biotec) according to the manufacturer’s protocol. Leukocyte isolation was performed with 40% / 80% discontinuous Percoll gradient (GE Healthcare). Cells were incubated with FcγR blocking reagent (rat anti-mouse CD16/CD32, BD Biosciences) for 30 minutes on ice prior to incubation with the other antibodies. Antibodies used for flow cytometry analysis are listed in S1 Table. To detect expression of Std fimbriae, flow cytometry was performed as previously described [16] with modifications. In brief, approximately 5x10^8^ bacteria were fixed with 10% formalin and incubated at room temperature for 20 minutes. After washing with PBS, cells were resuspended in 2% NGS diluted in PBS and incubated at room temperature for 30 minutes. Polyclonal rabbit anti-StdA serum [38] was added to the cell suspensions following incubation at room temperature for 30 min. After washing with PBS, fluorescently labeled secondary antibodies (Invitrogen) were added. Flow cytometry was performed using a MACSQuant Analyzer 10 (Miltenyi Biotec). The data were analyzed using FlowJo v.10 software (TreeStar).

### ELISA

Supernatants from the organ homogenates were collected and stored at −20°C. Lipocalin-2 levels were detected using mouse lipocalin-2/NGAL DuoSet ELISA (R&D Systems) according to the manufacturer’s protocol. Absorbance was measured using a Synergy HTX microplate reader and acquired using Gen5 software (Biotek).

### Western blot

*E*. *coli* StdOFF and *E*. *coli* StdON were grown in LB broth supplemented with carbenicillin (50 μg/ml) and to induce *std* fimbrial expression anhydrotetracycline (100 ng/ml) at 37°C until an OD_600_ of 0.6 was reached. 10^8^ bacteria were pelleted, resuspended in PBS, mixed with an equal volume of Laemmli buffer supplemented with 10% DTT, and boiled for 10 min. These whole-cell lysates were spun down and supernatants were loaded immediately onto a SDS-PAGE gel (15%). Proteins were transferred to Hybond-P 0.45 PVDF (Amersham) membranes using a Trans-Blot semi-dry transfer cell (Bio-Rad). After blocking with Roti-block (Carl Roth), membranes were incubated first with anti-StdA [38] serum (Humphries et al., 2003) diluted 1:500 in blocking buffer, and then with a goat anti-rabbit-HRP conjugate, and finally with Pierce ECL Western Blotting Substrate (Thermo Fisher Scientific). Images were obtained using the ImageQuant LAS 4000 system (GE Healthcare).

### *Salmonella* gene expression *in vivo*

Feces of the infected *Fut2*^+/+^ and *Fut2*^-/-^ mice (day 7 p.i.) was immediately stored in RNAlater (Ambion). Total RNA was extracted using the High Pure RNA Tissue Kit (Roche) and reverse transcription was performed with the cDNA Synthesis Kit (Roche) in accordance with the manufacturer’s instructions. Quantitative real-time PCR (qPCR) was performed on a CFX96 Real-Time PCR Detection System (Bio-Rad) using the Power SYBR Green PCR Master Mix (Applied Biosystems). Gene-specific primers are listed in S2 Table. Data were normalized to the house-keeping gene *rpoD* and analyzed by ΔΔCt method [50] with median values of *Fut2*^+/+^ mice infected with *S*. Tm Δ*aroA* as calibrators.

### Statistical analyses

All data were analyzed using GraphPad Prism V7.0d software. Statistical analyses were performed using one-way analysis of variance followed by Tukey’s multiple comparison test or Wilcoxon-Mann-Whitney test as indicated. Graphs display the mean values ± SD, unless stated otherwise. Competitive index data were analyzed by the Wilcoxon signed-rank test by comparing medians with a hypothetical value of 1.

## Supporting information

S1 Table Antibodies and lectins used in this study. (DOCX)

S2 Table Primers used in this study. (DOCX)

S1 Fig *Fut2*-deficient mice are more susceptible to *Salmonella* infection on day 1 p.i. Streptomycin-treated mice were infected with WT *S*. Typhimurium SL1344 or *S*. Typhimurium Δ*aroA* and sacrificed at day 1 p.i. (A) *S*. Typhimurium loads were determined in the cecum by plating homogenates on LB agar with streptomycin (n = 4–5 mice per group). Higher bacterial loads were observed in *Fut2*^*-/-*^ mice infected with wildtype *Salmonella*. (B) Histology scoring revealed similar levels of inflammation in both *Fut2*^*+/+*^ and *Fut2*^*-/-*^ ceca at day 1 p.i. (C) H&E staining of cecum tissue sections at 1 day p.i. Scale bars, 50 μm. Ceca of *Fut2*^*+/+*^ and *Fut2*^*-/-*^ mice were characterized by high numbers of cells in the lumen (L), increased numbers of inflammatory cells in mucosa (M), massive epithelial cell desquamation, and the formation of submucosal edema (E) upon *S*. Typhimurium wildtype and Δ*aroA* infection. (D) Similar cecum weights in both *Fut2*^*+/+*^ and *Fut2*^*-/-*^ mice were observed (n = 4–5 mice per group). *p<0.05; n.s = not significant, Mann-Whitney test.
(TIF)

S2 Fig *Fut2^+/+^* mice carry higher bacterial burden at later infection time points. Streptomycin-treated *Fut2*^*+/+*^ and *Fut2*^*-/-*^ mice were infected with *S*. Typhimurium Δ*aroA* for 3 and 14 days. (A-C) *S*. Typhimurium loads were determined in cecum tissue, cecum content and colon by plating homogenates on LB agar supplemented with streptomycin (n = 4–8 mice per group). (D) Lipocalin-2 levels measured by ELISA in supernatants of cecum tissues homogenates (n = 5) were higher in *Fut2*^*+/+*^ mice comparing to *Fut2*^*-/-*^ mice (day 7 p.i.). * P<0.05; n.s. = not significant, Mann-Whitney test.
(TIF)

S3 Fig Immune cells in murine colon tissue after *Salmonella* infection at day 7 p.i. Similar frequencies of (A) CD19^+^, (B) CD11c^+^ and (C) CD3^+^CD8^+^ cells were detected in colonic lamina propria of both *Fut2*^*+/+*^ and *Fut2*^*-/-*^ using flow cytometry (n = 6 mice per group). n.s. = not significant, Mann-Whitney test.
(TIF)

S4 Fig Std fimbriae mediate adhesion to intestinal epithelial cell culture in a fucose-dependent manner. (A) Caco-2 cells were infected with *E*. *coli* ORN172 expressing Std fimbriae (StdON) or not (empty vector). UEA-I (green), F-actin (purple) and StdA (red) staining of formalin-fixed Caco-2 cells displaying StdA-expression by StdON strain (top) and different degrees of fucosylation by Caco-2 cells. Scale bars, 20 μm. (B) *E*. *coli* ORN172 overexpressing Std (StdON) exhibited higher adherence to differentiated Caco-2 cells compared to *E*. *coli* ORN172 (empty). Adherence of *E*. *coli* StdON was abrogated upon addition of UEA-I lectin but not by addtion of DBA lectin prior to infection. **p<0.002; n.s. = not significant, ANOVA with Tukey’s multiple comparison test. (C) Imaging of Std fimbriae on *E*. *coli* ORN172 StdON by atomic force microscopy (AFM). The height profile is indicated by heatmaps. Insert show 2.5-fold enlarged details of the cell envelope.
(TIF)

S5 Fig Bacterial gene expression *in vivo*. *Salmonella* gene expression in feces of *Fut2*^*+/+*^ and *Fut2*^*-/-*^ mice infected with either *S*. Typhimurium Δ*aroA* or *S*. Typhimurium Δ*aroA*Δ*stdA* was measured. Gene expression was normalized to *rpoD*. Comparable levels of *stdA* expression was observed in both *Fut2*^*+/+*^ and *Fut2*^*-/-*^ mice infected with *S*. Typhimurium Δ*aroA* (A). Neither *fucI* (B) nor *pduBC* (C) transcription was affected by *Fut2* genotype or the presence or absence of *stdAB* genes. No significant differences were detected using one-way ANOVA with Tukey’s post test.
(TIF)

S6 Fig Std fimbriae are differentially expressed *in vivo* on day 1 p.i. *Fut2*^+/+^ and *Fut2*^*-/-*^ mice (n = 5 of each genotype) were infected with the reporter strain *S*. Typhimurium *stdA*stop::*gfp*. Mice were sacrificed on day 1 p.i. and colon sections were stained with anti-GFP antibody to detect GFP-positive bacteria. Std(GFP)-expressing *S*. Typhimurium were found mostly in lumen of colon, and only few bacteria expressed Std within mucosa. Scale bars, 10 μm.
(TIF)

S7 Fig Std producing *Salmonella in vivo* on day 7 p.i. stained with anti-Std antiserum. Colon sections of *Fut2*^+/+^ and *Fut2*^*-/-*^ mice were stained with an anti-*Salmonella* antibody (green). A subset of *Salmonella* stained positive with anti-Std antiserum (red). Fucosylation was visualized with UEA-1 lectin staining (grey) and nuclei were stained with DAPI (blue). Scale bars, 10 μm.
(TIF)

S8 Fig Deletion of *stdAB* genes does not affect motility and growth of S. Typhimurium. (A) *S*. Typhimurium SL1344 WT and *S*. Typhimurium SL1344 Δ*stdA* are similar in terms of motility. Deletion of *stdAB* genes has no effect on bacterial growth rate in WT (B) and Δ*aroA* (C) backgrounds.
(TIF)

S9 Fig Competitive index in feces. Competitive index (CI) was determined by infecting *Fut2*^*+/+*^ and *Fut2*^*-/-*^ mice (n = 10 per group) with an equal amount of *S*. Typhimurium Δ*aroA* and *S*. Typhimurium Δ*aroA*Δ*stdA*. Fecal homogenates from day 1, 3, 5 p.i. of both *Fut2*^*+/+*^ and *Fut2*^*-/-*^ mice were plated on LB agar containing streptomycin (total *Salmonella*) and on LB plates with streptomycin+kanamycin (*S*. Typhimurium Δ*aroA*Δ*stdA* only). Wilcoxon signed-rank test, p values are indicated.
(TIF)
