# Supplementary material for: Std fimbriae-fucose interaction increases Salmonella-induced intestinal inflammation and prolongs colonization
Source: PLoS Pathog. 2019 Jul 22;15(7):e1007915. doi: 10.1371/journal.ppat.1007915 (PMC6675130; doi:10.1371/journal.ppat.1007915)
Supplement: S1 Table — (DOCX) [file ppat.1007915.s001.docx]

**S1 Table. Antibodies and lectins used in this study.**

| **Antibody** | **Reference/Manufacturer, RRID and catalog number** |
| --- | --- |
| Polyclonal rabbit anti-StdA serum | Humphries et al., 2003 |
| F(ab)2-Goat anti-Rabbit IgG (H+L) Cross-Adsorbed Secondary Antibody, HRP | Thermo Fisher Scientific Cat# A10547, RRID:AB_2534046 |
| CD3 antibody | Abcam Cat# ab5690, RRID:AB_305055 |
| CD68 antibody [KP1], prediluted | Abcam Cat# ab845, RRID:AB_306567 |
| Anti-Myeloperoxidase (Mature / Immature Myeloid Cell Marker) Ab-1 Polyclonal Antibody, Unconjugated | Lab Vision Cat# RB-373-A, RRID:AB_59597 |
| F(ab)2-Goat anti-Mouse IgG (H+L) Cross-Adsorbed Secondary Antibody, Alexa Fluor 633 | Thermo Fisher Scientific Cat# A-21053, RRID:AB_2535720 |
| F(ab)2-Goat anti-Rabbit IgG (H+L) Cross-Adsorbed Secondary Antibody, Alexa Fluor 546 | Thermo Fisher Scientific Cat# A-11071, RRID:AB_2534115 |
| CD16/CD32 antibody | BD Biosciences Cat# 553142, RRID:AB_394657 |
| APC/Cyanine7 anti-mouse CD45 antibody | BioLegend Cat# 103116, RRID:AB_312981 |
| APC anti-mouse CD4 antibody | BioLegend Cat# 100411, RRID:AB_312696 |
| PE/Cy7 anti-mouse Ly-6G antibody | BioLegend Cat# 127618, RRID:AB_1877261 |
| CD3 Monoclonal Antibody (17A2), FITC, eBioscience(TM) | Thermo Fisher Scientific Cat# 11-0032-80, RRID:AB_2572430 |
| CD11c Monoclonal Antibody (N418), FITC, eBioscience(TM) | Thermo Fisher Scientific Cat# 11-0114-82, RRID:AB_464940 |
| CD8a Monoclonal Antibody (53-6.7), PE, eBioscience(TM) | Thermo Fisher Scientific Cat# 12-0081-81, RRID:AB_465529 |
| F4/80 Monoclonal Antibody (BM8), PerCP-Cyanine5.5, eBioscience(TM) | Thermo Fisher Scientific Cat# 45-4801-82, RRID:AB_914345 |
| CD11b Monoclonal Antibody (M1/70), PE, eBioscience™ | Thermo Fisher Scientific Cat# 12-0112-82, RRID:AB_2734869 |
| Rat Anti-Mouse Ly-6C Monoclonal Antibody, APC Conjugated, Clone AL-21 | BD Biosciences Cat# 560595, RRID:AB_1727554 |
| PerCP-Cy™5.5 Rat Anti-Mouse CD19 Clone 1D3 | BD Biosciences Cat# 561113, RRID:AB_10563071 |
| Purified anti-*E. coli* RNA Sigma 70 antibody | BioLegend Cat# 663202, RRID:AB_2564410 |
| Polyclonal rabbit anti-*E. coli* antibody | Abcam Cat# ab137967 |
| Anti-CD324 (E-Cadherin) Alexa Fluor® 647 100 ug antibody | Thermo Fisher Scientific Cat# 51-3249-82, RRID:AB_1210532 |
| Green fluorescent protein (GFP) antibody - DSHB; University of Iowa | DSHB Cat# DSHB-GFP-12A6, RRID:AB_2617417 |
| *Salmonella* O Antiserum Group B Factors 1, 4, 12, 27 | BD Difco Cat# 229731 |
| F(ab)2-Goat anti-Mouse IgG (H+L) Cross-Adsorbed Secondary Antibody, Alexa Fluor 488 | Thermo Fisher Scientific Cat# A-11017, RRID:AB_2534084 |
| **Lectin** | **Manufacturer, RRID and catalog number** |
| Ulex europaeus [UEA-I] (FITC) lectin | CosmoBIO Cat# CAC-JOM-J519, accession #P22972 |
| Wheat germ agglutinin [WGA] (Rhodamine) lectin | Vector Laboratories Cat# RL-1022, RRID:AB_2336871 |
| Dolichos biflorus agglutinin [DBA] (FITC) lectin | Vector Laboratories Cat# FL-1031, RRID:AB_2336394 |
