# Supplementary material for: Std fimbriae-fucose interaction increases Salmonella-induced intestinal inflammation and prolongs colonization
Source: PLoS Pathog. 2019 Jul 22;15(7):e1007915. doi: 10.1371/journal.ppat.1007915 (PMC6675130; doi:10.1371/journal.ppat.1007915)
Supplement: S2 Table — (DOCX) [file ppat.1007915.s002.docx]

**Table S2.** Primers used in this study.

| **Primer** | **Sequence (5' to 3')** | **Description** |
| --- | --- | --- |
| gNRAMP1 fw s | CAGCATCCCGCTGTGGGA | *NRAMP1* genotyping |
| gNRAMP1 fw r | CAGCATCCCGCTGTGGGG |  |
| gNRAMP1 re | ACAGCCCGGACAGGTGGG |  |
| rpoD-fw | GGTCTGACCATCGAACAGGTG | *rpoD* housekeeping gene |
| rpoD-re | ATCAGACCGATGTTGCCTTC |  |
| stdA-fw | CGG CTG CCG GTA TGA TGT | *stdA* gene expression |
| stdA-rw | GGG CCT GCT GTG GGT GTA |  |
| STm fucI-fw | CGCCGCCTGTGAAGAAAAAT | *fucI* gene expression |
| STm fucI-re | GTAAACCGCCCCAGGACGCT |  |
| pduBC-F | CACCAGCTTTAGTAACGAAGC | *pduBC* genes expression;  Fuchs and Staib, 2015 |
| pduBC-R | CAAAGCCGTCCTGATTCAC |  |
