# Supplementary figures and images for: Std fimbriae-fucose interaction increases Salmonella-induced intestinal inflammation and prolongs colonization
Source: PLoS Pathog. 2019 Jul 22;15(7):e1007915. doi: 10.1371/journal.ppat.1007915 (PMC6675130; doi:10.1371/journal.ppat.1007915)

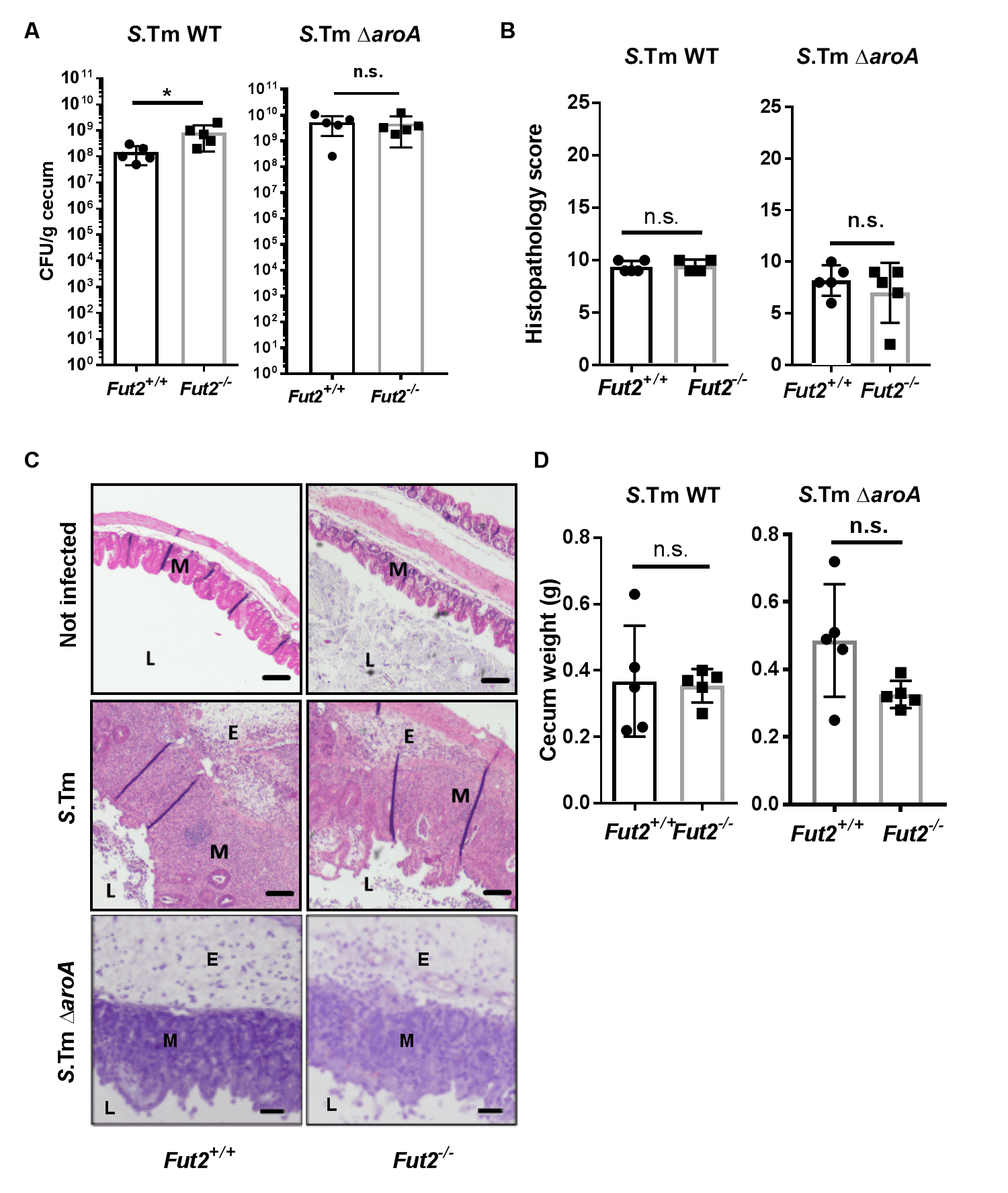

Supplement: S1 Fig — Streptomycin-treated mice were infected with WT S. Typhimurium SL1344 or S. Typhimurium ΔaroA and sacrificed at day 1 p.i. (A) S. Typhimurium loads were determined in the cecum by plating homogenates on LB agar with streptomycin (n = 4–5 mice per group). Higher bacterial loads were observed in Fut2-/- mice infected with wildtype Salmonella. (B) Histology scoring revealed similar levels of inflammation in both Fut2+/+ and Fut2-/- ceca at day 1 p.i. (C) H&E staining of cecum tissue sections at 1 day p.i. Scale bars, 50 μm. Ceca of Fut2+/+ and Fut2-/- mice were characterized by high numbers of cells in the lumen (L), increased numbers of inflammatory cells in mucosa (M), massive epithelial cell desquamation, and the formation of submucosal edema (E) upon S. Typhimurium wildtype and ΔaroA infection. (D) Similar cecum weights in both Fut2+/+ and Fut2-/- mice were observed (n = 4–5 mice per group). *p<0.05; n.s = not significant, Mann-Whitney test. (TIF) [file ppat.1007915.s003.tif]

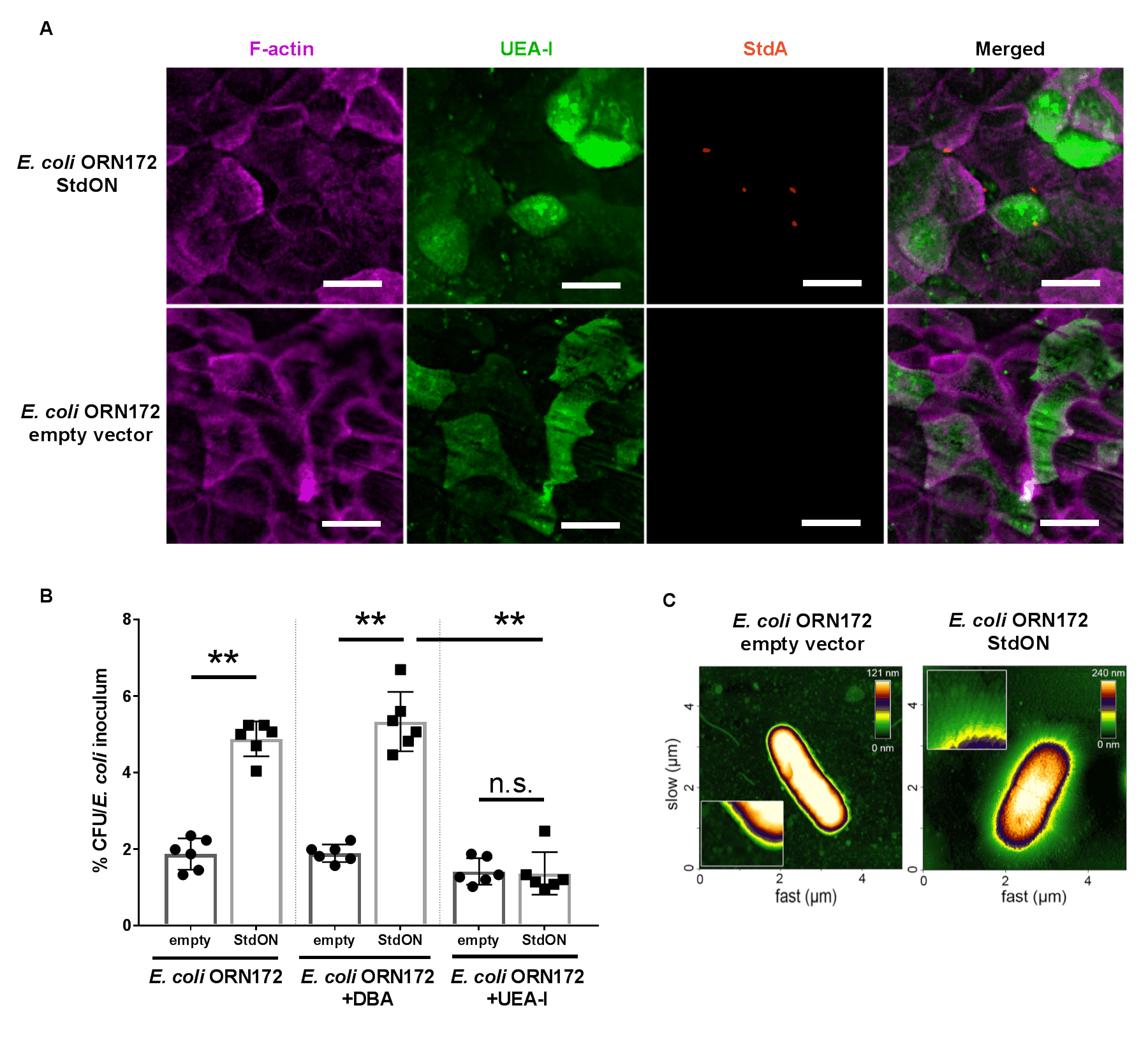

Supplement: S4 Fig — (A) Caco-2 cells were infected with E. coli ORN172 expressing Std fimbriae (StdON) or not (empty vector). UEA-I (green), F-actin (purple) and StdA (red) staining of formalin-fixed Caco-2 cells displaying StdA-expression by StdON strain (top) and different degrees of fucosylation by Caco-2 cells. Scale bars, 20 μm. (B) E. coli ORN172 overexpressing Std (StdON) exhibited higher adherence to differentiated Caco-2 cells compared to E. coli ORN172 (empty). Adherence of E. coli StdON was abrogated upon addition of UEA-I lectin but not by addtion of DBA lectin prior to infection. **p<0.002; n.s. = not significant, ANOVA with Tukey’s multiple comparison test. (C) Imaging of Std fimbriae on E. coli ORN172 StdON by atomic force microscopy (AFM). The height profile is indicated by heatmaps. Insert show 2.5-fold enlarged details of the cell envelope. (TIF) [file ppat.1007915.s006.tif]

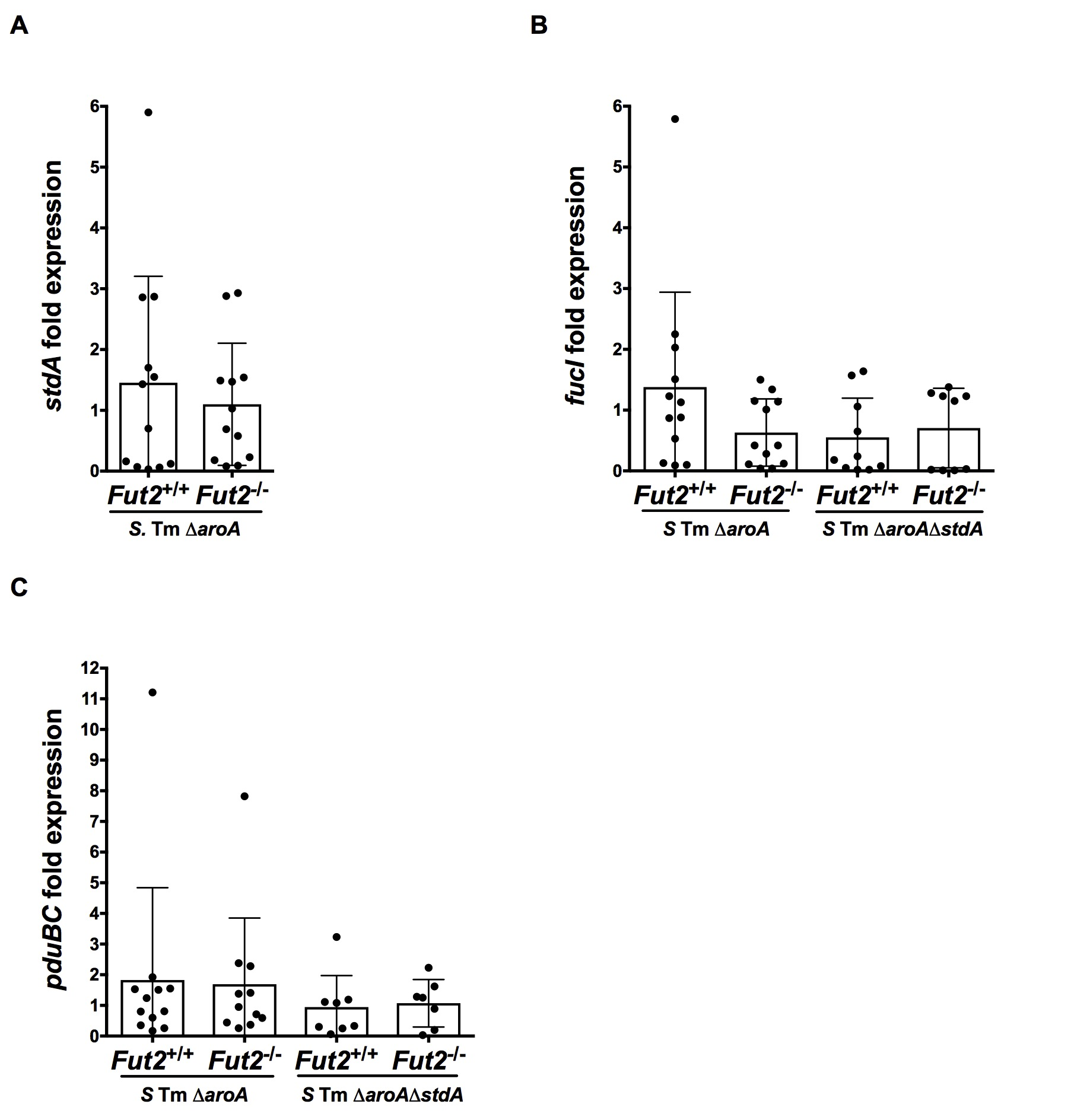

Supplement: S5 Fig — Salmonella gene expression in feces of Fut2+/+ and Fut2-/- mice infected with either S. Typhimurium ΔaroA or S. Typhimurium ΔaroAΔstdA was measured. Gene expression was normalized to rpoD. Comparable levels of stdA expression was observed in both Fut2+/+ and Fut2-/- mice infected with S. Typhimurium ΔaroA (A). Neither fucI (B) nor pduBC (C) transcription was affected by Fut2 genotype or the presence or absence of stdAB genes. No significant differences were detected using one-way ANOVA with Tukey’s post test. (TIF) [file ppat.1007915.s007.tif]

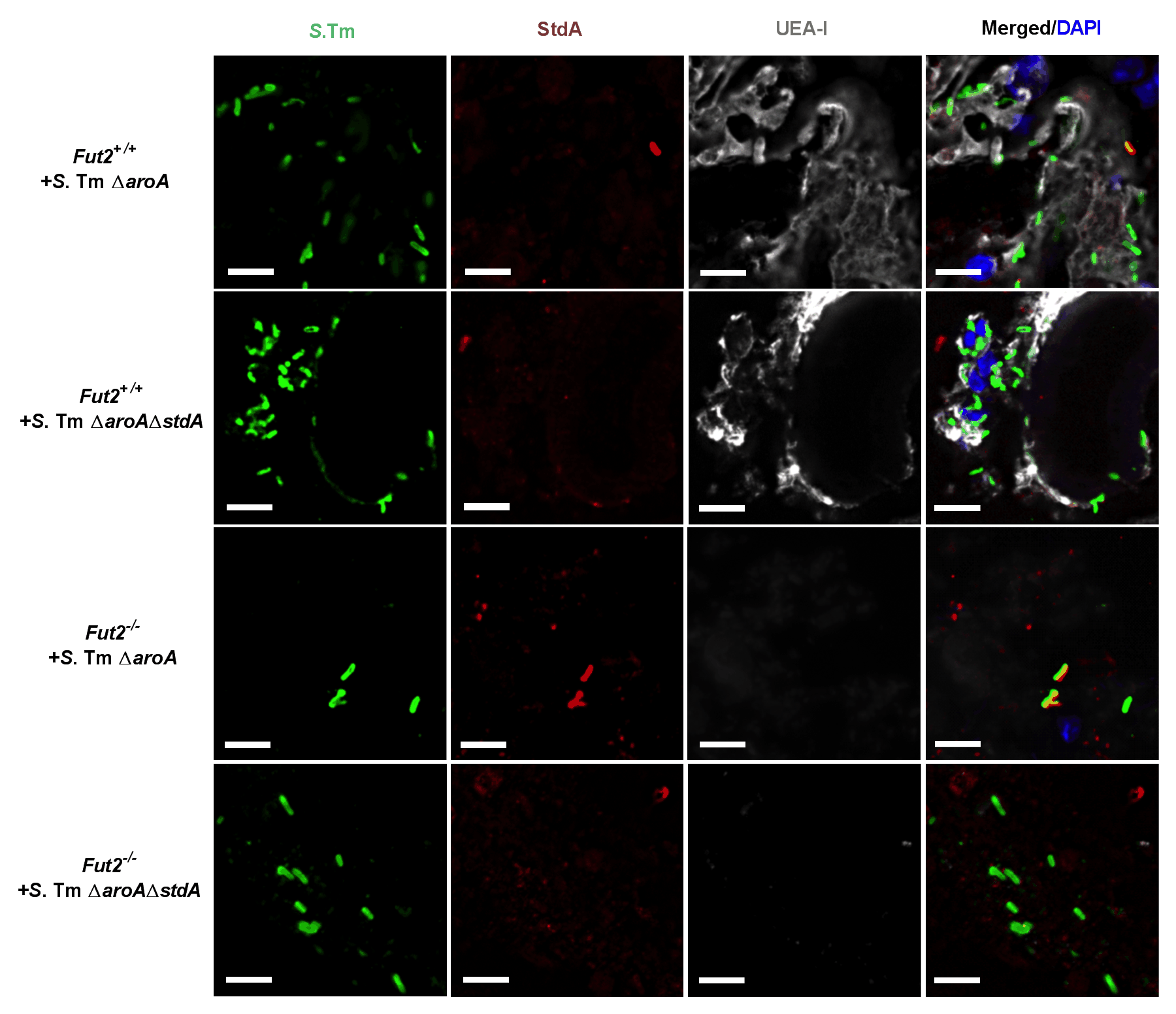

Supplement: S7 Fig — Colon sections of Fut2+/+ and Fut2-/- mice were stained with an anti-Salmonella antibody (green). A subset of Salmonella stained positive with anti-Std antiserum (red). Fucosylation was visualized with UEA-1 lectin staining (grey) and nuclei were stained with DAPI (blue). Scale bars, 10 μm. (TIF) [file ppat.1007915.s009.tif]

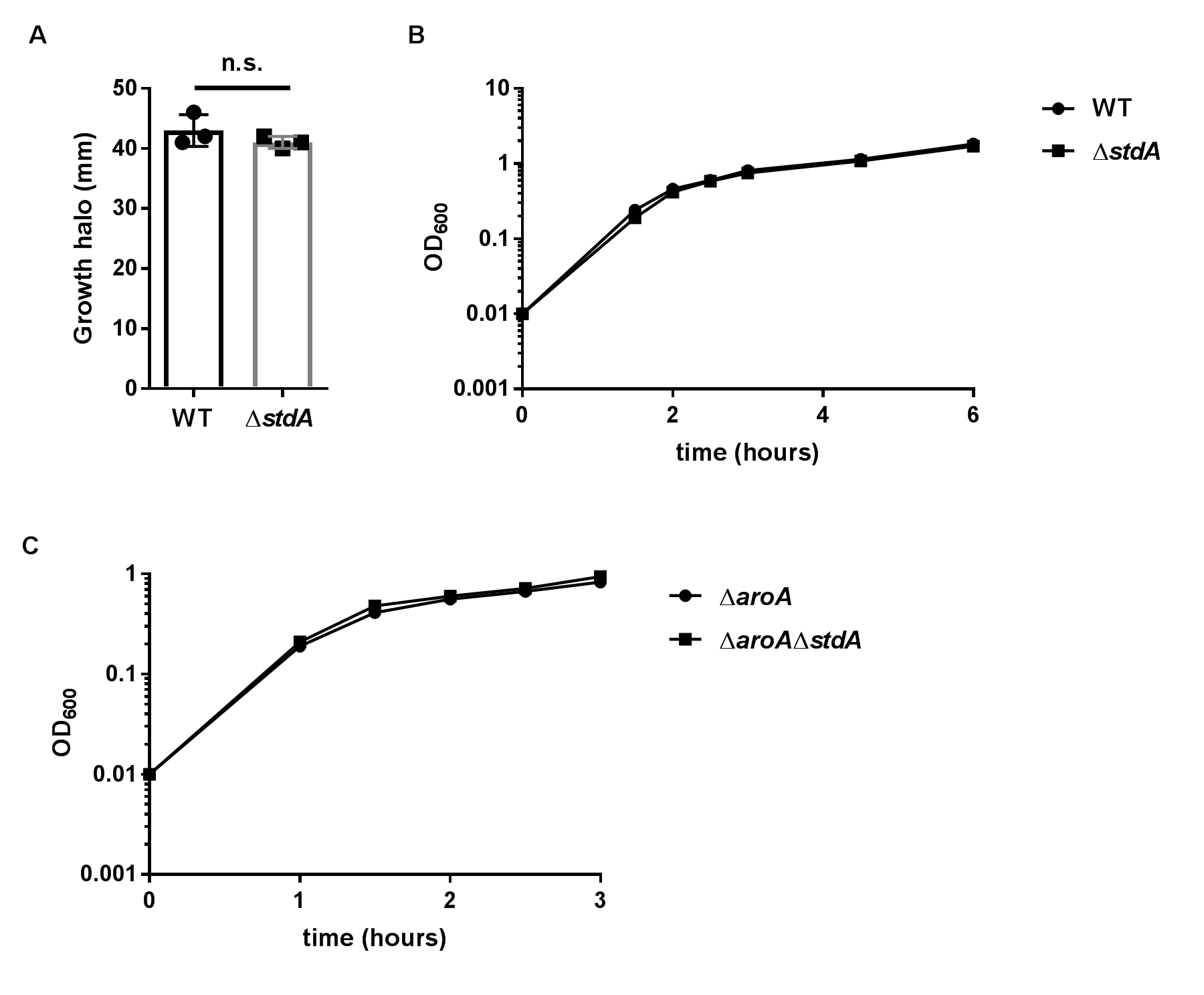

Supplement: S8 Fig — (A) S. Typhimurium SL1344 WT and S. Typhimurium SL1344 ΔstdA are similar in terms of motility. Deletion of stdAB genes has no effect on bacterial growth rate in WT (B) and ΔaroA (C) backgrounds. (TIF) [file ppat.1007915.s010.tif]

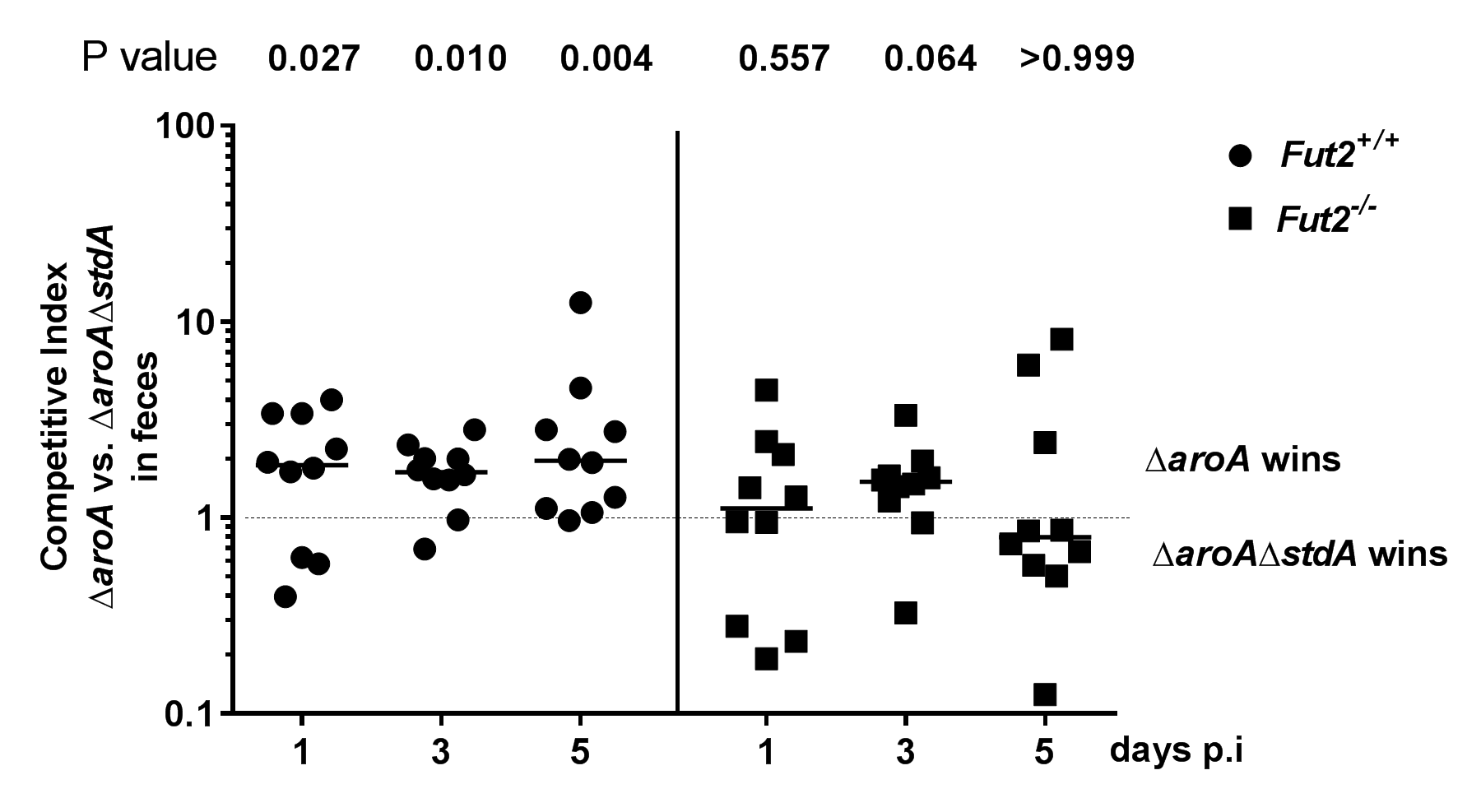

Supplement: S9 Fig — Competitive index (CI) was determined by infecting Fut2+/+ and Fut2-/- mice (n = 10 per group) with an equal amount of S. Typhimurium ΔaroA and S. Typhimurium ΔaroAΔstdA. Fecal homogenates from day 1, 3, 5 p.i. of both Fut2+/+ and Fut2-/- mice were plated on LB agar containing streptomycin (total Salmonella) and on LB plates with streptomycin+kanamycin (S. Typhimurium ΔaroAΔstdA only). Wilcoxon signed-rank test, p values are indicated. (TIF) [file ppat.1007915.s011.tif]
